# The Acetyltransferase RibT From *Bacillus subtilis* Affects *in vivo* Dynamics of the Multimeric Heavy Riboflavin Synthase Complex

**DOI:** 10.3389/fmicb.2022.856820

**Published:** 2022-04-14

**Authors:** Daniel Andreas Orlando Rotter, Christoph Heger, Christian Kühm, Nina Schmidt, Antje Schäfer, Thomas Heimerl, Matthias Mack, Peter L. Graumann

**Affiliations:** ^1^SYNMIKRO, Center for Synthetic Microbiology, Philipps-Universität Marburg, Marburg, Germany; ^2^Department of Chemistry, Philipps-Universität Marburg, Marburg, Germany; ^3^BioNTech Manufacturing Marburg GmbH, Marburg, Germany; ^4^BioSpringBiotechnolgie GmbH, Frankfurt am Main, Germany; ^5^Institute of Technical Microbiology, University of Applied Sciences Mannheim, Mannheim, Germany; ^6^Department of Biology, Philipps-Universität Marburg, Marburg, Germany

**Keywords:** acetyltransferase, GNAT, riboflavin, RibT, single particle tracking, heavy riboflavin synthase, *Bacillus subtilis*, post-translational modification

## Abstract

Flavins are ubiquitous molecules in life as they serve as important enzyme cofactors. In the Gram-positive, soil-dwelling bacterium *Bacillus subtilis*, four well-characterized gene products (the enzymes RibDG, RibE, RibAB, and RibH) catalyze the biosynthesis of riboflavin (RF) from guanosine-triphosphate (GTP) and ribulose-5-phosphate (R5P). The corresponding genes form an operon together with the gene *ribT* (*ribDG-E-AB-H-T*), wherein the function of this terminal gene remained enigmatic. RibT has been structurally characterized as a GCN5-like acetyltransferase (GNAT), however, with unidentified target molecules. Bacterial two-hybrid system revealed interactions between RibT, RibH, and RibE, forming the heavy RF synthase complex. Applying single particle tracking (SPT), we found that confined (sub)diffusion of RibT is largely dependent on interacting RibE and, to a lesser degree, on interacting RibH. By induced expression of otherwise low-expressed *ribT* from an ectopic locus, we observed a decrease in the subpopulation considered to represent capsids of the heavy RF synthase and an increase in the subpopulation thought to represent pentamers of RibH, pointing to a putative role for RibT in capsid disassembly. Complementarily, either deletion of *ribT* or mutation of a key residue from RibH (K29) suspected to be the substrate of RibT for acetylation leads to increased levels of subpopulations considered as capsids of RibH-mVenus (RibH-mV) in comparison to wild-type (wt)-like cells. Thus, we provide evidence for an indirect involvement of RibT in RF biosynthesis by a putative capsid disassembling mechanism considered to involve acetylation of RibH residue K29 at the three-fold symmetry axis of 60-mer capsids.

## Importance

Regulation of metabolism is crucial for all organisms and can occur at many different levels, including modulation of enzyme levels and of their activity. Additionally, some pathways include substrate channeling, adding a further level of complexity for possibilities of adapting product formation to changing needs of cells. Enzyme activity can be strongly affected by chemical, reversible modification of polypeptide chains. We show that Acetyltransferase RibT affects riboflavin synthesis in *Bacillus subtilis*, which is also used for industrial production of the vitamin. RibT interacts with both enzymes, forming the substrate channeling heavy riboflavin (RF) synthase, RibH, and RibE, and acetylates a key residue on RibH, which forms the large outer shell of the synthase. We show that, surprisingly, RibT activity affects the dynamics of the 60 (+3)-mer heavy RF synthase complex, as well as its structure, likely *via* changing the assembly/disassembly equilibrium. Changes in single-particle dynamics affect the subcellular distribution of the enzyme complex, whose accumulation at subpolar regions of cells is altered. Thus, an acetyltransferase can alter subcellular specificity of enzymes and directly or indirectly modulate product formation of a metabolic pathway.

## Introduction

Post-translational modifications (PTMs) are ubiquitous among all domains of life and offer a fast and simple way to alter functions of cellular proteins by the enzymatic introduction of small modifications at key residues. Besides phosphorylation, which is the most prominent PTM involved, e.g., in signal transduction, acetylation of proteins is also increasingly recognized as an important, prevalent, and dynamic PTM in bacteria like for the Gram-positive, soil-dwelling bacterium *Bacillus subtilis* (*B. subtilis*) (Carabetta et al., 2016, 2019; Carabetta and Cristea, 2017). Enzymatic acetylation is catalyzed by a conserved protein superfamily termed general control non-repressed protein 5 (GCN5)-like acetyltransferases (GNATs), which usually utilize Acetyl-Coenzyme A (AcCoA) as an acetyl-moiety donor for transfer onto a variety of different acceptor substrates. A regulatory and reversible enzymatic acetylation had been first discovered in eukaryotes where histone acetyltransferases (HATS) serve to remodel the chromatin structure in the nucleus. Acetylation of surface-exposed lysine residues from histone proteins neutralizes their positive charges, thereby decreasing their affinity to the phosphate backbone of chromosomal DNA substantially and thus finally resulting in transcriptional activation by allowing the access of transcription factors and RNA polymerases. Being the antagonist for HATS, Histone deactylases catalyze the reverse reaction, causing the typical nucleosome structure of DNA tightly wrapped around octameric histones as a consequence of histone deacetylation. Intriguingly, in bacteria like *B. subtilis*, where nucleoid and intracellular proteome are not physically separated by specialized compartments, the specific GNAT YfmK similarly regulates nucleoid compaction by acetylating a nucleoid-associated protein (NAP), termed Histone-like protein (HBsu) at multiple sites (Carabetta et al., 2019). Moreover, regulation of cell wall growth when cells transit from exponential to the stationary growth phase in *B. subtilis* has been shown to be impacted by differential acetylation of the shape-determining protein MreB by an unknown GNAT (Carabetta et al., 2016). Until now, YfmK and AcuA are the only functionally characterized GNATs out of 48 GNAT domain-containing proteins in *B. subtilis*, indicating a large potential for further regulatory roles of acetylation (Gardner and Escalante-Semerena, 2008; Zhu and Stulke, 2018). Interestingly, a phyloproteomic analysis suggests that acetylation of key catalytic residues in enzymes from primary metabolism (the glycolysis and tricarboxylic acid cycle) is widely conserved among distant-related bacteria, underlining a general regulatory role for the activity of GNATs in metabolism (Nakayasu et al., 2017).

From genomic data, it has been inferred that, in the Gram-positive soil-dwelling bacterium *B. subtilis*, ~1.5% of all proteins depend on the riboflavin-derived cofactors flavin mononucleotide (FMN) and flavin adenine dinucleotide (FAD), mainly oxidoreductases (around 90%) and, to a minor extent, also enzymes catalyzing non-redox reactions like transferases, lyases, isomerases, and ligases (Macheroux et al., 2011). The four well-characterized genes essential for biosynthesis of FMN/FAD precursor RF (*ribDG, ribE, ribAB, ribH*) are organized in one operon (*rib*-operon), including a terminal fifth gene (*ribT* or *ypzK* in older nomenclatures). The RibT protein was shown to be a potential GNAT (EC No. 2.3.1.-), but its role in RF metabolism remained unclear. Contrary to the polycistronic mRNA comprising the entire *rib*-operon, for which expression is regulated by an FMN riboswitch (Mironov et al., 2002; Winkler et al., 2002), transcription of *ribT* mRNA has been shown to be additionally driven by flavin-independent, constitutive expression from its own promotor, indicating cellular importance for flavin-independent regulation of RibT levels (Mironov et al., 1994; Nicolas et al., 2012; Sklyarova et al., 2012). Interestingly, a patent for RF overproducing *B. subtilis* strains described a decrease in RF production upon deletion of a genetic region containing *ribT*, indicating an indirect involvement in RF biosynthesis (Perkins et al., 1991). Previously, we have characterized diffusion, interplay, and localization of RF biosynthesis (Rib) enzymes in *B. subtilis* (Rotter et al., 2021). So far, the respective gene product RibT has also been crystalized with CoA, but, surprisingly, not with AcCoA, however, with unidentified acceptor molecule(s) (Srivastava et al., 2018). From a proteomic approach investigating the acetylome in *B. subtilis*, we found the high-abundance enzyme lumazine synthase (RibH; ~3,600–4,000 copies) reported to be acetylated at primary sequence position K29 (Carabetta et al., 2016). Intriguingly, three of these residues originating from three pentamers occupy each center of the 20 three-fold symmetry axes from RibH capsids (Supplementary Figure S1), considered to represent putative binding sites for the encapsulated RF synthase (RibE) (Han and Woycechowsky, 2017). Those capsids are built from 12 pentameric RibH molecules, encapsulating a trimer of RibE, which is then termed heavy RF synthase (Ladenstein et al., 1988). However, the underlying mechanism governing encapsulation of RibE *in vivo* is largely unknown (Bacher et al., 1986). Recently, we have found, in accordance with previous *in vitro* data, that not all enzymes present are forming heavy RF synthases *in vivo*; rather, there is an existing equilibrium between fully assembled units and freely diffusive RibH pentamers as well as free RibE trimers (Bacher et al., 1980; Rotter et al., 2021). Our results indicate that RibT affects SPT dynamics of the heavy RF synthase complex by a potential posttranslational mechanism involving acetylation of RibH at residue K29.

## Results

### RibT Interacts With the Two Partner Proteins RibH and RibE Forming Heavy RF Synthase and With Itself

**Figure 1:**
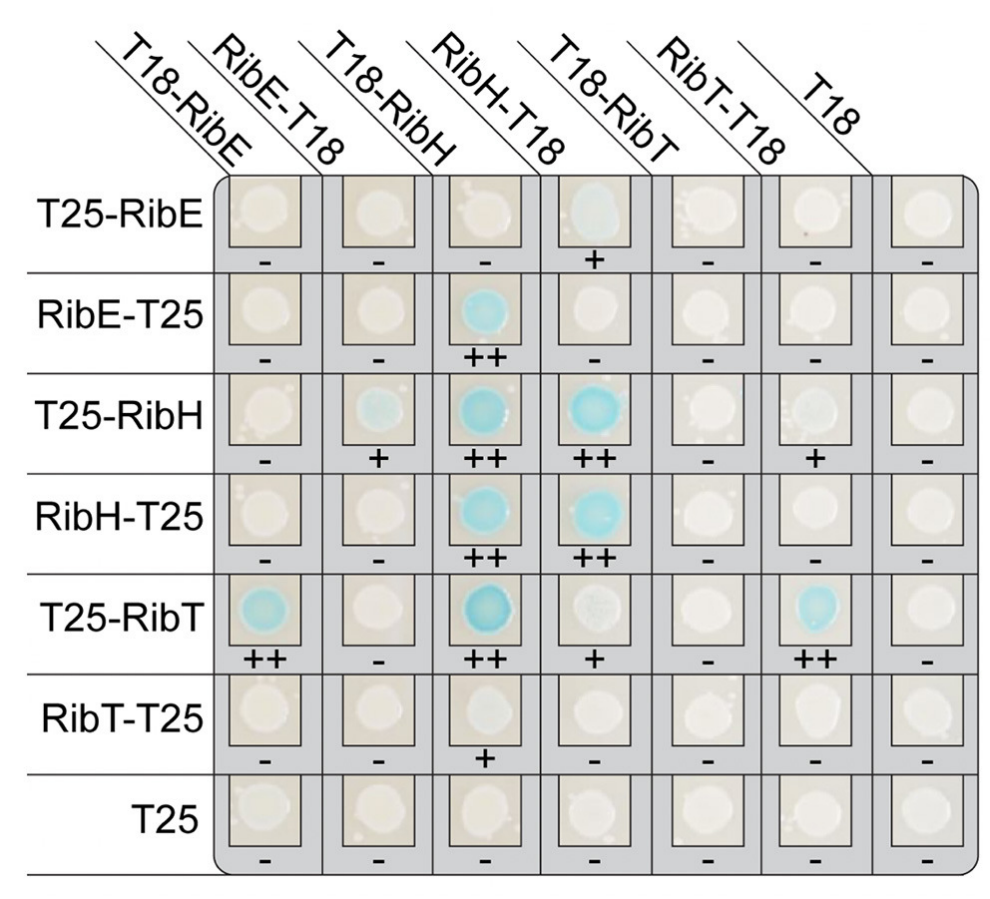
Results of bacterial adenylate cyclase two-hybrid system (BACTH) in *E. coli* reporter strains using domain fusions to the Rib enzymes. The coproduced fusion enzymes are indicated on top and the respective partner fusion protein on the left. The scoring below each colony indicates the level of blue coloring, ranging from – (none), over + (light), to ++ (clearly visible).

A bacterial two-hybrid analysis was carried out to shed light on the possible interaction between RibT and the two partner proteins of the heavy RF synthase complex, RibE and RibH. The hypothesis was that the acetyltransferase RibT interacts with these two proteins and transfers acetyl groups. Plasmid constructs containing the coding regions of each of the full-length enzymes RibT, RibE, and RibH were fused to the coding regions of the N- and C-terminus of the T18 fragment of adenylate cyclase. Similarly, plasmid constructs containing the same coding regions were also fused to the coding regions of the N- and C-terminus of the T25 fragment of adenylate cyclase. *E. coli* strains were transformed with two different plasmids, and all possible combinations between the Rib enzymes were tested. An interaction between the Rib enzymes would bring together the T18 and T25 fragments of adenylate cyclase-generating cAMP, which, in turn, would lead to expression of the *lacZ* reporter gene with its activity measured qualitatively on plates. Figure 1 shows one result from three different experiments (other two shown in Supplementary Figure S2).

As already known, we found interactions between RibH and RibE (Figure 1). RibE is known to exist as trimer, formed by interactions of C-terminal helices from each monomer, and occurs either solely or encapsulated in the cytoplasm (Bacher et al., 1980; Rotter et al., 2021). Furthermore, the C-terminus has been described to contain a localization tag required for encapsulation by RibH (Han and Woycechowsky, 2017). Interactions of RibH with RibE could be observed for three of eight possible cases (T18-RibH with RibE-T25, RibH-T18 with T25-RibE, as well as with RibE-T25).

Intriguingly, we also found interactions between RibE and RibT, as well as between RibH and RibT (Figure 1). Moreover, we found interaction between RibT itself, indicating possible dimer formation. Similarly, all fusions to RibH resulted in self-interaction. As expected, not all C- and N-terminal combinations of other fusion proteins revealed interactions: in case for self-interaction of RibT, we found interaction for the N-terminal fusion T25-RibT with the C-terminal fusion RibT-T18 only, but no interaction was detected for the N-terminal fusion T25-RibT with the other N-terminal fusion protein T18-RibT. This indicates either a spatial hindrance by the fused domains, shielding the monomers from self-interaction or by a distance that is too large between the split adenlylate cyclase, preventing generation of an active enzyme. Similarly, all other C-terminal fusion of T25-RibT completely abolished interaction with every protein shown, whereas the N-terminal fusion T25-RibT showed interactions for three cases (T18-RibT, T18-RibH, and RibT-T18), highlighting the importance of the C-terminus of RibT for the interactions found. It is of note that we did not find any interactions between RibT domain fusions and any Rib enzymes (RibAB or RibDG) other than RibE or RibH (results not shown), supporting that RibT acts specifically on RibH and RibE.

### RibT Reveals Bipartite Diffusion in the Cytoplasm Characteristic for Binding Events to the Rib Enzymes RibH and RibE

**Figure 2:**
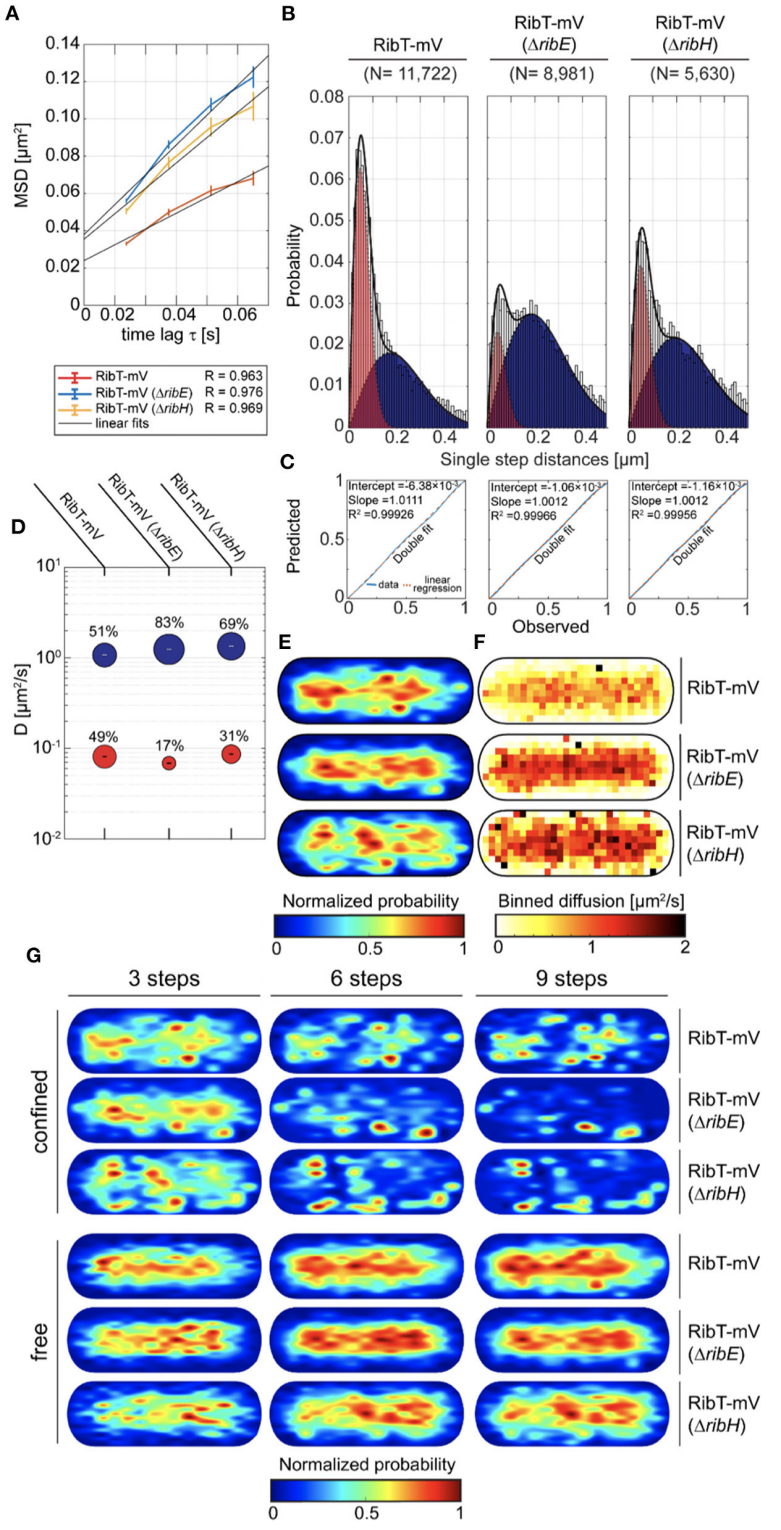
Cytoplasmic diffusion of RibT is bipartite, revealing free and highly confined motion. **(A)** EAMSD vs. a time plot for RibT-mV either in the presence of RibH and RibE or with *ribH* or *ribE* gene being deleted. **(B)** JD analysis of RibT-mV, RibT-mV (Δ*ribH*), and RibT-mV (Δ*ribE*) SPT data using a two-component Rayleigh Mixture model. **(C)** Model prediction vs. observation plots for the Rayleigh Mixture models of JDs in **(B)**. **(D)** Bubble plots displaying relative proportions of subpopulations for the three strains with RibT-mV analyzed and their according DCs that are derived from JD analysis in **(B)**. **(E)** Spot-location heat-maps displaying the spatial distributions of trajectories for RibT-mV in the absence or presence of RibE or RibH. Trajectories were projected into a normalized cell, which resembles an averaged sized cell of *B. subtilis* in the mid-exponential growth phase (1 μm ×3 μm). The likelihood of finding trajectories at a certain place in the cytoplasm is indicated by a color code from blue to red (indicated below). Signal intensities of spatial distribution maps have been normalized with each other. **(F)** Speed map representations of RibT-mV either in the presence or absence of RibH or RibE displaying the spatial distributions of single-step displacements binned over areas of.1 μm^2^ for normalized cells. **(G)** Subcellular analysis of confined and free trajectories for all three strains with RibT-mV is shown for different numbers of consecutive steps projected onto the cytoplasm of a normalized cell representation. Displayed are the results using the radii given in Table 3 with three, six or nine consecutive steps of confinement. The likelihood of finding trajectories at a certain place in the cytoplasm is indicated by a color code from blue to red (indicated below). Signal intensities of spatial distribution maps have been normalized with each other.

Knowing the interacting enzymes of RibT, we were interested in its localization and diffusion in *B. subtilis* using SPT under RF-producing conditions. For this purpose, we created a construct encoding for a C-terminal mVenus (mV) fluorescent protein fusion to the *ribT* gene, which was subsequently integrated into the original locus of the chromosome, such that its expression is driven from its native promotor(s). The presence of full-length RibT-mV has been verified by In-Gel fluorescence detection from cell lysates of the respective strain (Supplementary Figure S3). In order to study if interactions between RibT and RibH with RibE affect RibT-mV dynamics, we created mutant strains in which either the *ribH* or the *ribE* gene is substituted by an antibiotic resistance cassette (Koo et al., 2017). RF auxotrophy was addressed by the supplementation of RF to culture media for those two deletion strains. We first analyzed SPT datasets for all three strains by ensemble-averaged mean squared displacement (EAMSD). We found that overall average diffusion of RibT-mV (0.21 μm^2^/s) was increased by the deletion of either *ribH* (0.34 μm^2^/s) or *ribE* (0.4 μm^2^/s) (Figure 2A; Table 1), indicating that disruptions of interactions increase the number of freely diffusing RibT molecules. To determine potential diffusive subpopulations and their respective diffusion coefficients, we used a multiple Rayleigh Mixture Model-based approach for analysis of Jump distances (JD), derived from SPT trajectories. This way, we found that RibT-mV shows two diffusive states, a freely diffusive state characterized by diffusion in the range of 1.1–1.35 μm^2^/s and more than ten-fold lower diffusive states in the range of ~0.07-0.09 μm^2^/s (Figure 2B), indicating motion together with a large complex. Figure 2C shows that a two-population fit explains the observed data very well. In wt-like cells, the distribution of the two populations was almost equal, with 51% diffusing with 1.1 μm^2^/s and 49% diffusing with 0.08 μm^2^/s (Figure 2D; Table 1). However, deletion of either *ribE* or *ribH* leads to a shift in JD distributions toward increased diffusion (Figure 2B). The ratio of subpopulations relative to the wt-like condition changed such that, in case of deletion of *ribE*, the high-diffusing subpopulation increased to 83% (accounting for a relative increase of ~63%), or to 69% (accounting for a relative increase of ~35%) in the *ribH* deletion (Figure 2D). Accordingly, the low-diffusing subpopulation strongly decreased to 17% (diffusing with ~0.07 μm^2^/s) in *ribE* mutant cells, whereas deletion of *ribH* leads to a decrease in 31% in subpopulation size (diffusing with ~0.09 μm^2^/s), which accounts for relative decreases of ~65 and 37%, respectively (Figure 2D; Table 1). Note that the *ribT* gene has its own promoter, and fluorescence signal intensity of RibT-mV was not reduced in mutant cells.

**Table 1:** A summary of DCs determined by EAMSD or JD analysis.

**Strain relevant genotype**	**Diffusion (μm^**2**^/s) from EAMSD (R-value)**	**D_**1**_ (μm^**2**^/s) (Pop. %)**	**D_**2**_ (μm^**2**^/s) (Pop. %)**	**D_**3**_ (μm^**2**^/s) (Pop. %)**	**N of cells (N of movies)**	**N of trajectories per cell (N of total trajectories)**	**Av. life time (s) (frames)**	**Av. cell length (μm)**
*ribT-mV*	0.21 (0.963)	0.081 ± 0 (48.7)	1.07 ± 0.003 (51.3)	–	170 (70)	10 (1,792)	0.1 (7.6)	2.67
*ribT-mV (*Δ*ribE)*	0.40 (0.976)	0.069 ± 0 (16.6)	1.24 ± 0.001 (83.4)	–	96 (37)	16.5 (1,524)	0.095 (6.9)	2.90
*ribT-mV (*Δ*ribH)*	0.34 (0.969)	0.087 ± 0 (31.4)	1.35 ± 0.003 (68.6)	–	106 (32)	9 (955)	0.095 (6.9)	3.06
*ribH-mV*	0.34 (0.997)	0.087 ± 0 (12.1)	0.37 ± 0 (65)	1.05 ± 0.001 (22.9)	129 (39)	129 (16,084)	0.14 (10)	2.92
*ribH-mV* (Δ*ribT*)	0.34 (0.997)	0.08 ± 0 (8.16)	0.39 ± 0 (71.6)	1.09 ± 0.001 (20.2)	202 (68)	88 (16,261)	0.14 (9.9)	2.66
*ribH-mV* (*amyE*::*P_*Xyl*_ribT*)	0.43 (0.996)	0.35 ± 0.001 (37.8)	0.87 ± 0.001 (62.3)	-	97 (30)	166 (15,607)	0.13 (9.5)	2.78
*ribH^*K*29*R*^-mV*	0.34 (0.998)	0.078 ± 0 (10.7)	0.37 ± 0 (70.1)	1.08 ± 0.001 (19.3)	132 (54)	143 (17,641)	0.15 (11)	2.92
*ribH^*K*29*E*^-mV*	0.34 (0.996)	0.048 ± 0 (10.2)	0.35 ± 0 (60.7)	0.97 ± 0.001 (29.1)	190 (59)	96 (17,416)	0.13 (9.8)	2.89

To study localization of RibT-mV, we created spot location heat-maps derived from all trajectories and projected those into normalized cells, resembling *B. subtilis* cells in the exponential growth phase (Figure 2E). We found that RibT-mV is distributed in the entire cytoplasm, which represents a tube-like structure (Figure 2E). When free and confined (molecules staying within a confined area for more than a predefined number of steps) motion was analyzed separately, we found that free diffusion of RibT-mV is found throughout the center of the cell, whereas confined diffusion is mostly found toward the periphery of the cytoplasm, including polar regions (Figure 2G). The area of confinement was determined according to the localization error of each dataset (Supplementary Figure S4; **Table 3**).

Deletion of *ribH* or of *ribE* leads to increased diffusion over central regions, as indicated by our speed-map analysis (Figure 2F). Comparing the relative amounts of static trajectories that are confined (at least three time intervals) revealed that deletion of *ribE* leads to a decrease in ~32%, from ~41% in wt-like cells, whereas the deletion of *ribH* had smaller impact on the percentage of static trajectories (~38%, Supplementary Table S1). For longer times of molecule confinement (six and nine time intervals), this trend continued, showing ~20% (~9% for nine time intervals) static trajectories of RibT-mV in wt cells, whereas deletion of *ribE* leads to a decrease to ~10% (~4% for nine time intervals), and deletion of *ribH* to a decrease in ~15% (~7% for nine time intervals) of static tracks. Because strong confinement leads to subdiffusion, we analyzed confined trajectories separately by pooling them for EAMSD analysis, including statistical significance tests for their mode of diffusion (Supplementary Figure S5). Using six time lags, we found that subdiffusion is only evident for the wt-like strain having no deletions, whereas deletion of either *ribE* or *ribH* leads to entirely Brownian motion (Supplementary Figure S5A). When nine time lags were considered for analysis, we found that deletion of *ribE* changed the mode of diffusion for RibT-mV from subdiffusive to Brownian, whereas deletion of *ribH* or wt-like conditions revealed subdiffusion, as well (Supplementary Figure S5B). Thus, the deletion of *ribE* and of *ribH* affects diffusion of RibT-mV. Overall, our results from SPT suggest that RibT-mV binds both partner proteins of the heavy RF synthase *in vivo*.

### Ectopic Expression of RibT Changes Diffusion of RibH-mV, Suggesting an Involvement in Capsid Dissassembly

Because RibT-mV SPT dynamics are affected by deletion of the interacting gene products, we next examined how RibH-mV motion is affected by the absence or higher abundance of RibT. When we analyzed a non-fluorescent strain in which *ribT* was deleted, we could only find a very small effect on exponential growth using minimal media (Supplementary Figure S6). This finding indicates that the deletion of *ribT* does not affect RF yield such that this becomes severely growth limiting. Nevertheless, we measured a decrease in RF yield upon deletion of *ribT* (Supplementary Figure S6), indicating that the gene product optimizes RF biosynthesis *in vivo*.

**Figure 3:**
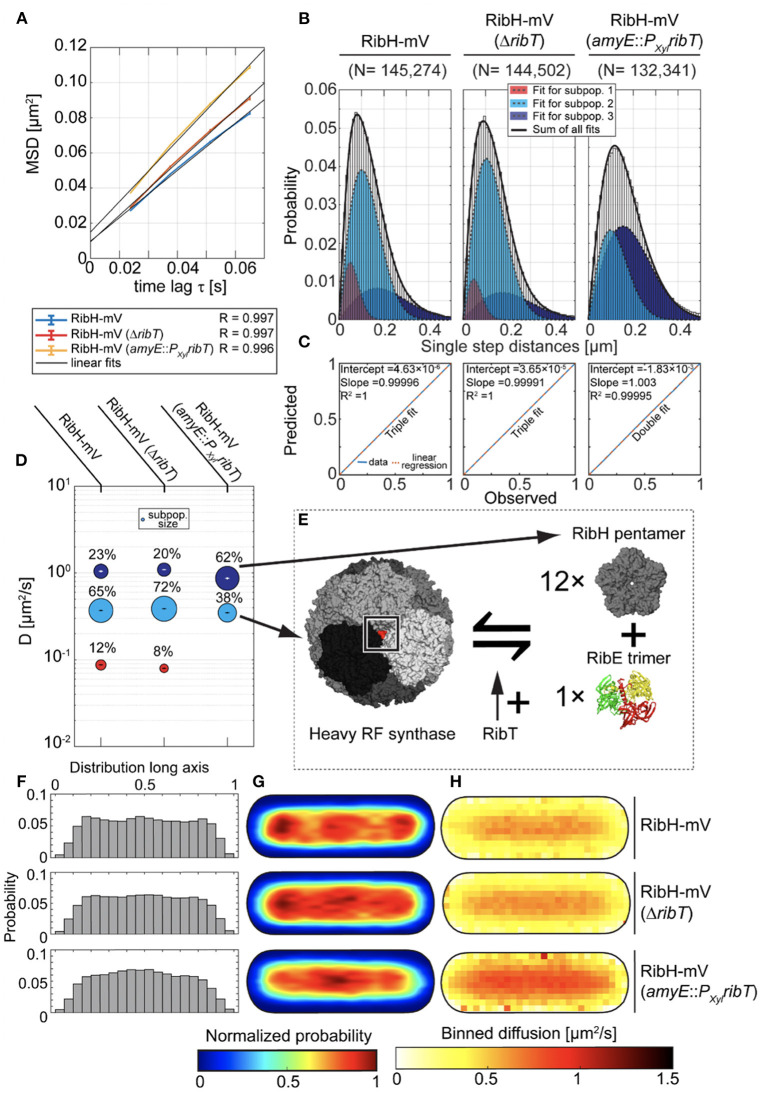
Production of RibT from an ectopic locus in *B. subtilis* induces changes in diffusion of RibH-mV. **(A)** EAMSD vs. the time plot for three strains carrying RibH-mV either in the presence of *ribT* gene expressed from original locus (RibH-mV), or with *ribT* gene being deleted from the nucleoid (RibH-mV, Δ*ribT*), or with *ribT* gene being expressed from an inducible, ectopic gene locus (RibH-mV, *amyE*::*P*_*Xyl*_*ribT*). **(B)** JD analysis of RibH-mV and RibH-mV (Δ*ribT*) SPT data using a three-component Rayleigh Mixture model, as well as RibH-mV (*amyE::P*_*Xyl*_*ribT*) using a two-component Rayleigh Mixture model. **(C)** Model prediction vs. observation plots for the Rayleigh Mixture models of JDs in **(B)**. **(D)** Bubble plots displaying relative proportions of subpopulations for the three strains with RibH-mV analyzed and their according DCs that are derived from JD analysis in **(B)**. **(E)** A model explaining how RibT is thought to affect the equilibrium between fully assembled capsids and free pentamers. **(F)** Histograms displaying the normalized probabilities of finding trajectories along the long axis of cells for the three different strains. **(G)** Spot location heat-maps displaying the spatial distributions of trajectories for the three different strains carrying RibH-mV. Trajectories were projected onto the two-dimensional cytoplasmic area of a normalized cell, which resembles an averaged sized cell of *B. subtilis* in the mid-exponential growth phase (1 μm × 3 μm). The likelihood of finding trajectories at a certain place in the cytoplasm is indicated by a color code from blue to red (indicated below the cell maps). Signal intensities of spatial distribution maps have been normalized with each other. **(H)** Speed-map representations of RibH-mV displaying the spatial distributions of single-step diffusion binned over areas of 0.1 μm^2^ for normalized cells. The color code (from white to red) indicating the values for binned diffusion is given below the speed-map representations.

When we compared EAMSD plots for RibH-mV from wt-like cells (DC of ~0.34 μm^2^/s), we found little change in comparison to cells having *ribT* deleted (DC of ~0.38 μm^2^/s) (Figure 3A). However, induced overexpression of *ribT* from an ectopic locus resulted in an overall increased diffusion (~0.43 μm^2^/s), and thus higher mobility of RibH-mV. In order to analyze datasets at the level of diffusive subpopulations, we further compared them by JD analysis. In case for the *ribT* deletion, we found three existing diffusive subpopulations for RibH-mV resembling those of the wt-like strain (Figure 3B). Here, the triple fit yielded better results than the double fit (Figure 3C). As suggested before (Rotter et al., 2021), the medium-mobile subpopulation best agrees with comprising capsids of RibH-mV, and was increased by ~11% in the absence of RibT, relative to the wt cells (Figure 3D). Accordingly, the fast-mobile subpopulation thought to consist of RibH-mV pentamers (Figure 3E), diffusing with ~0.87 μm^2^/s −1.05 μm^2^/s, is relatively decreased by ~13% for the *ribT* deletion strain. Furthermore, the lowest diffusing subpopulation is also decreased (8% subpopulation size) for the *ribT* deletion strain in comparison to the wt-like strain (12% subpopulation size) (Figure 3D), which would account for a relative decrease in the confined subpopulation of 33%. Strikingly, we found no such low diffusive subpopulation for RibH-mV when *ribT* was expressed ectopically (Figure 3D). However, we found a large increase in the subpopulation thought to represent pentamers (62% subpopulation size), diffusing with 0.87 μm^2^/s, relative to wt-like cells, and a respective decrease in the fast-mobile subpopulation (38% subpopulation size), diffusing with 0.35 μm^2^/s. The change of the putative pentamer fraction is, therefore, quite striking in comparison to wt cells with a relative increase of 169.5%, whereas the change of the capsid subpopulation accounts for a relative decrease of 41.5%. These results indicate that RibT changes dynamics of the heavy RF synthase quite remarkably. Changes in molecule speeds can be seen in Figure 3H. RibH-mV is accumulated at the cell poles and in the cell middle (representing the future cell poles), and thus excluded from nucleoids (Rotter et al., 2021). This localization pattern is less pronounced in the absence of RibT and lost upon overproduction of RibT (Figure 3G, distribution quantified in Figure 3F). This effect was more evident when we further differentiated trajectories according to their behavior being classified as confined or freely diffusive (Supplementary Figure S7); confinement at polar regions is less pronounced for shorter events (three steps) and more so for six to nine steps.

Our findings can be best explained by an increase of assembled capsids and a decrease in pentamers by the deletion of *ribT*. Furthermore, the subpopulation considered as capsids showed decreased polar confinement for shorter times (three steps) compared to wt-like conditions, while the deletion of *ribT* had a noticeable positive effect on capsid abundance as judged from a relative increase of ~11% in comparison to wt-like cells. Thus, our findings indicate a role for RibT in capsid assembly and, more precisely, in increasing disassembly toward pentamers.

### RibH Is a Target for Acetylation by RibT *in vitro*

Residue K29 has been shown to be a target for acetylation (Carabetta et al., 2019), but the responsible acetyl transferase has not been identified. Therefore, we aimed at analyzing if K29 of RibH is a substrate for acetylation carried out by RibT. We employed a fluorescence-based detection assay in order to show the activity toward RibH. The assay is based on the detection of a fluorescence adduct that is formed upon reaction with free CoA in solution. Thus, the amount of free (fluorescent) CoA is proportional to the number of acetylated amine groups, and, thereby, the activity of any acetyl transferase utilizing AcCoA as a donor molecule can be measured indirectly by fluorescence readout at the appropriate wavelength (RibH^K29^-${\text{NH}}_{3}^{+}$ + RibT + Ac-S-CoA < -> RibH^K29^-NHAc + CoA-SH + H^+^+RibT, CoA-SH + dye -> fluorescent product).

We expressed RibH and mutant RibH^K29E^, which cannot be acetylated, as N-terminal Strep-tagged versions, as well as RibT as a C-terminal His-tagged protein (RibT-His_6_), using *E. coli* as heterologous system. Purification was done by two-step purification using Strep-tactin and size exclusion chromatography (SEC), which is shown for Strep-RibH as an example in Supplementary Figure S8. Similarly, we also purified RibT-His_6_ by Ni-NTA affinity chromatography and SEC (Supplementary Figure S9). In order to probe the importance of C112 for catalysis, we also created and tested activity of RibT^C112A^ as a His_6_-tagged version (RibT^C112A^-His_6_) toward RibH. As a control to prove the specificity of RibT toward K29 of RibH as a substrate, we made use of a mutant from RibH in which K29 is substituted by E, which cannot be acetylated due to the missing ε-amine group. Additionally, we applied bovine serum albumin (BSA) in similar concentrations as negative acceptor control.

**Figure 4:**
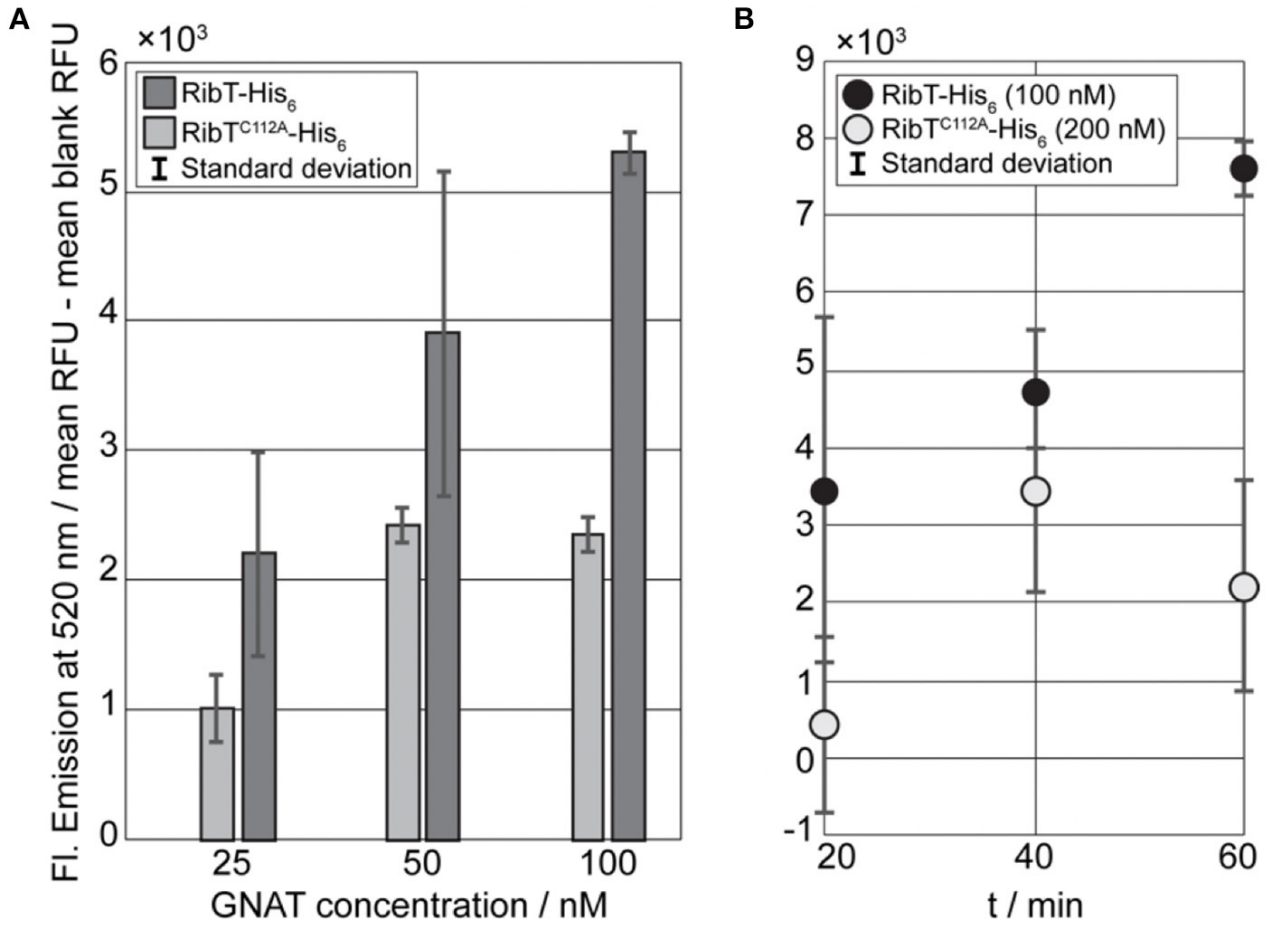
Fluorescence-based GNAT assays for the detection of RibT activity toward RibH. **(A)** Results of the GNAT titration end point assay using RibT-His_6_ and mutant RibT^C112A^-His_6_ in concentrations of 25 nM, 50 nM, and 100 nM, with a constant concentration of acceptor substrate Strep-RibH at 10 μM. Reactions were initiated by the addition of the GNAT enzyme to acceptor substrate and allowed to proceed at room temperature for 30 min until reactions were quenched, and resulting fluorescence was measured at 520 nm. All the samples prepared in duplicate standard deviation are shown within the bars. Prior to plotting, blank values were subtracted from measured sample values. **(B)** Kinetic GNAT end point assay using RibT-His_6_ (100 nM) and mutant RibT^C112A^-His_6_ (200 nM) with a fixed acceptor substrate concentration of 10-μM Strep-RibH. The reactions were initiated by the addition of the GNAT enzyme to acceptor substrate and allowed to proceed at room temperature for different time intervals, resulting fluorescence was measured at 520 nm. Prior to plotting, blank values had been subtracted from measured sample values.

In order to show qualitatively that acetylation occurs using RibH and RibT, we performed endpoint assays with varying concentrations (25, 50, and 100 nM) of RibT-His_6_ or mutant RibT^C112A^-His_6_ and a fixed concentration of acceptor protein Strep-RibH (10 μM; Figure 4A). We started the endpoint assay by addition of the GNAT to reaction mixtures and incubated reaction mixtures for 30 min at room temperature before these were quenched, and the fluorescence was read out at a microplate reader. We found that fluorescence increased with increasing GNAT concentrations, indicating GNAT activity of RibT-His_6_ toward Strep-RibH (Figure 4A). We also found activity toward Strep-RibH for the mutant RibT^C112A^-His_6_, however, much lower than for the wt enzyme; A two-fold decrease in GNAT activity is very likely the result of an increased dissociation constant for the substrate AcCoA (as also shown by Srivastava et al., 2018).

Next, we compared kinetics of acetylation using a kinetic endpoint assay for RibT-His_6_ and RibT^C112A^-His_6_, applying 10 μM of Strep-RibH as an acceptor. However, when using mutant RibT^C112A^-His_6_ with the same concentration (100 nM) as the non-mutated enzyme, we did not observe any detectable activity (results not shown). Therefore, we increased the concentration of the mutant GNAT by two-fold (200 nM) to ensure that the fluorescence signals derived from the reactions with free CoA do not fall below the detection range. We found that mutant RibT^C112A^-His_6_ has activity toward Strep-RibH, as well, but to a much lower extent than the wt enzyme (Figure 4B).

**Figure 5:**
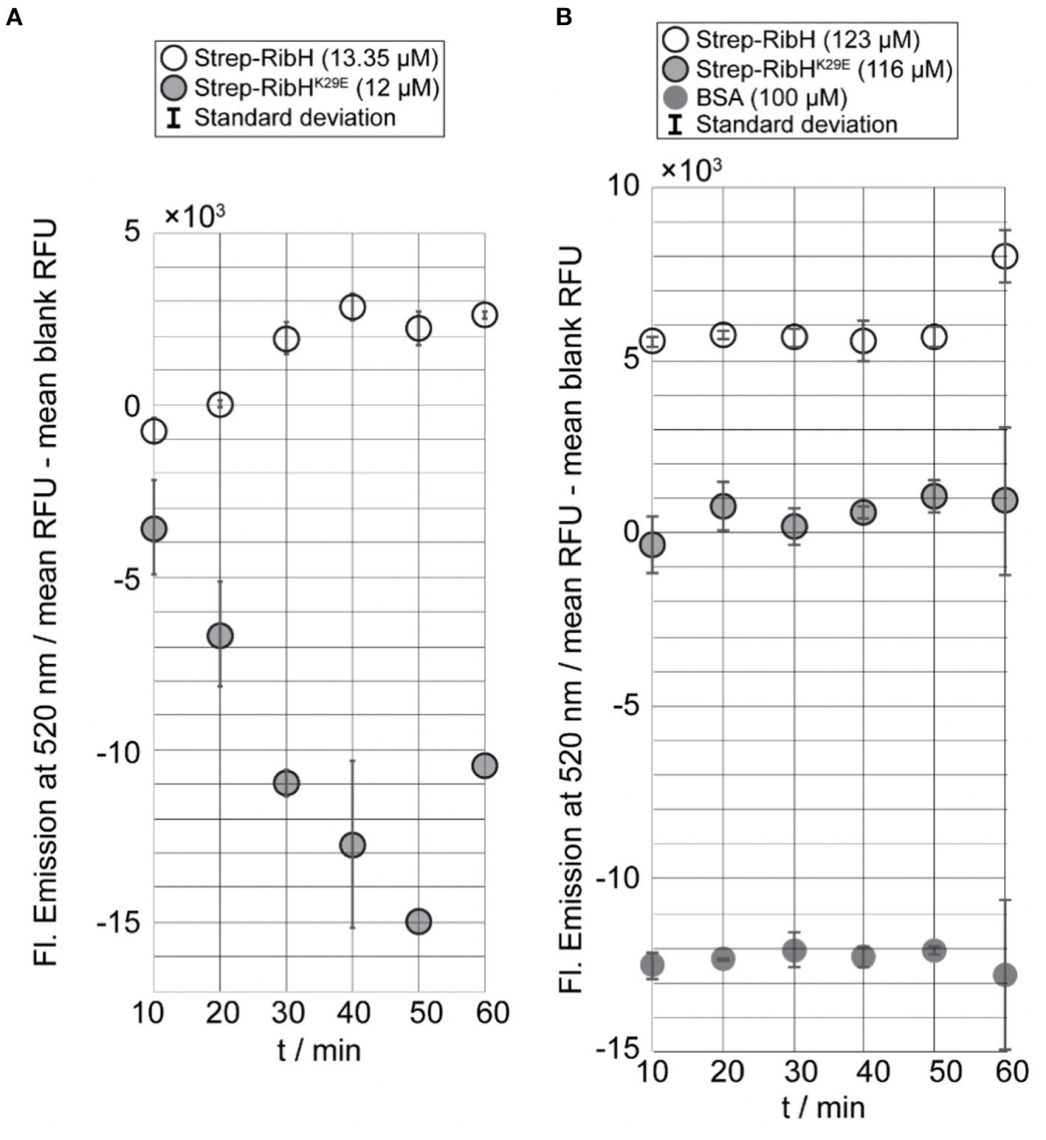
Kinetic end point assay using GNAT RibT-His_6_ and different concentrations of probed acceptor substrates and controls. Reactions were initiated by the addition of the GNAT enzyme to acceptor substrate and allowed to proceed at room temperature for the indicated time intervals (10 min) until reactions were quenched. After quenching fluorescence was developed, the samples were excited at 320 nm, and resulting fluorescence was measured at 520 nm. Prior to plotting, blank values had been subtracted from measured sample values. All the samples have been prepared in duplicate, and their respective standard deviation is shown within the bars (except for the last two data points of Strep-RibH^K29E^ when 12 μM was used because only one value was measured in both cases). **(A)** Results of kinetic end point assay using a constant concentration of RibT-His_6_ (100 nM) with Strep-RibH (13.25 μM) and Strep-RibH^K29E^ (12 μM). **(B)** Results of kinetic end point assay using a constant concentration of RibT-His_6_ (100 nM) with Strep-RibH (123 μM), Strep-RibH^K29E^ (116 μM), and BSA (100 μM).

From the kinetic end point assay, we can already conclude that RibH is, indeed, the acceptor molecule for acetylation by the GNAT RibT. Nevertheless, to prove its specificity for residue K29 in RibH, we made use of the mutant RibH^K29E^, which cannot be acetylated at this particular residue. Thus, we performed the kinetic end point assay using mutant Strep-RibH^K29E^, Strep-RibH, and BSA for two different concentration magnitudes (~10-123 μM) of acceptor probes using a fixed GNAT concentration of 100 nM. For the kinetic end point assay in which we used the lower acceptor concentration range, we found increasing fluorescence over time in case for Strep-RibH in linear dependency for the first four time increments (Figure 5A), underlining GNAT activity toward Strep-RibH. The later time points reveal a plateau, indicating that acetylation of Strep-RibH is completed after 40 min at this concentration. Contrarily, for mutant Strep-RibH^K29E^, which lacks the acceptor amino acid for acetylation, we observe a decrease in fluorescence over all time points, instead of an expected fluorescence distributed around zero (Figure 5A). Possibly, relatively high fluorescence values of the measured blank values lead to false-negative fluorescence after their subtraction.

At higher concentrations of acceptor substrates, and keeping GNAT concentrations constant at 100 nM, relative fluorescence of Strep-RibH^K29E^ did not increase over time, but, as expected for this mutant, was distributed around values of zero. In contrast, Strep-RibH showed a clearly increased relative fluorescence (~5- × -10^3^ RFU) for the first five time increments (Figure 5B). For the negative control BSA, we observed negative values for fluorescence (Figure 5B), because measured blank values were higher than analyzed samples. Similar to RibH^K29E^, fluorescence for BSA remained almost constant over time, strongly indicating no activity of RibT-His_6_ toward BSA. Thus, it is evident that Strep-RibH showed increased fluorescence in comparison to its mutant Strep-RibH^K29E^, or to BSA, indicating that RibH is, indeed, acetylated by RibT at K29.

### Acetylation-Mutants RibH^K29R^-mV and RibH^K29E^-mV Show Changes in Single-Particle Dynamics

**Figure 6:**
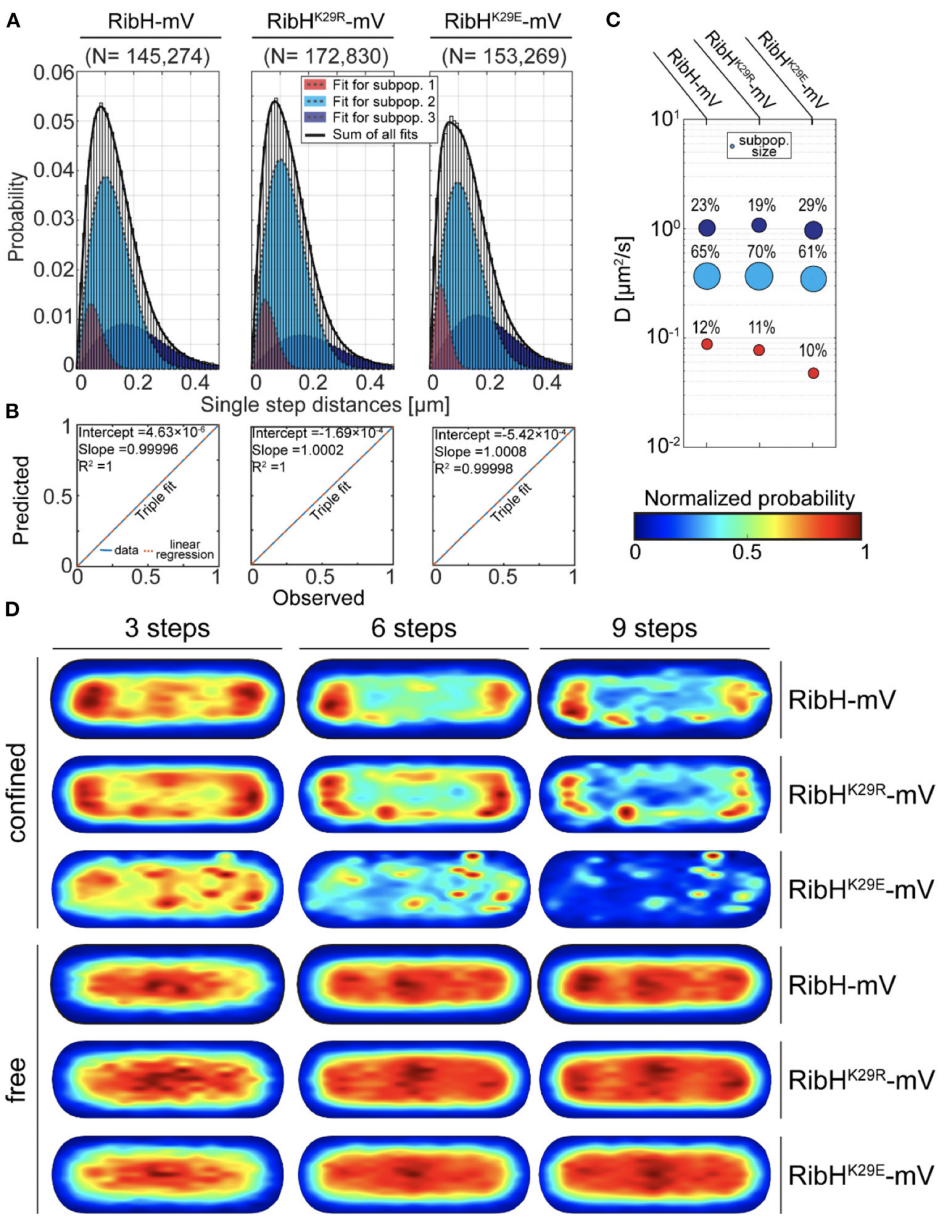
The RibH^K29R^-mV mutant reveals SPT dynamics similar to RibH-mV in absence of *ribT*, whereas the RibH^K29E^-mV mutant shows a decrease in the subpopulation considered to contain capsids. **(A)** JD analysis of RibH-mV SPT data in comparison with SPT datasets from two mutant strains RibH^K29R^-mV and RibH^K29E^-mV using a three-component Rayleigh Mixture model. **(B)** Model prediction vs. observation plots for the Rayleigh Mixture models of JDs in **(A)**. **(C)** Bubble plots displaying relative proportions of subpopulations for RibH-mV and the two mutant strains and their according DCs that are derived from JD analysis in **(A)**. **(D)** Subcellular analysis of confined and free trajectories shown for different numbers of consecutive steps projected onto the cytoplasm of a normalized cell representation. Displayed are the results using the radii of confinement given in Table 3 with three, six, or nine consecutive steps of confinement.

Because we found that changes in RibT levels have an effect on diffusive subpopulations of RibH-mV, we anticipated that the interaction between RibH and RibT, resulting in an acetylation event of RibH at residue K29, might change RibH dynamics. Thus, we visualized two mutants of RibH not capable of acetylation at the suspected residue (K29E and K29R); we hypothesized that mutation from K to R leads to mimicry of K that cannot be acetylated. We speculated that this change should result in a phenotype observed by SPT that resembles the phenotype of a *ribT* deletion strain, as presented before (Figure 3). On the other hand, mutant RibH^K29E^-mV introduces negative charges into the three-fold symmetry axis of the 60-meric capsids (Supplementary Figure S1), and might form less stable capsids due to the repulsive clash of the three negative charges. Overall DCs of mutants RibH^K29R^-mV (~0.34 μm^2^/s) and RibH^K29E^-mV (~0.34 μm^2^/s) by EAMSD analysis were found to be similar to RibH-mV (~0.34 μm^2^/s) (Table 1). However, analysis of JDs by multiple Rayleigh Mixture models resulted in three different subpopulations (Figures 6B,C), and showed that all three strains differed in their relative RibH proportions (Figure 6A). RibH^K29R^-mV revealed a relative increase of ~8% in the medium-mobile subpopulation considered as capsids (70% subpopulation size) in comparison to RibH-mV (65% subpopulation size), accompanied by a relative decrease of 24% in subpopulation considered as pentamers (19% subpopulation size) in comparison to the non-mutated strain (25% subpopulation size) (Figure 6C). Although these changes are small, they are significant because they are based on a very large data set. Comparison of trajectory localizations and visualization of diffusions using normalized cell representations for the acetylation mutants revealed that NO seen for the K29R mutant is comparable to that of the wt-like enzyme, and that diffusion is also distributed in a similar manner over the cytoplasm, being higher over central, nucleoid regions and lower toward the cytoplasmic periphery (Supplementary Figures S10A,B). Contrarily, for the K29E, mutant hotspots of trajectory localizations are visible in heat-maps and coincide with spaces of low diffusion, indicating that the K29E mutation leads to an altered localization (Supplementary Figures S10A,B).

When comparing subcellular localizations for trajectories classified as confined or freely diffusive (Figure 6D), the RibH^K29R^-mV mutant showed very similar distributions for both classes of trajectories compared to the wt-like enzyme, retaining a pattern of nucleoid occlusion (NO). Contrarily, the medium-mobile population decreased for RibH^K29E^-mV, while the high-mobile fraction increased (Figure 6C). Importantly, NO was lost for the mutant protein, best seen for six and nine steps of confined motion (Figure 6D). Thus, mutation of K29 leads to small but noticeable changes in single particle dynamics of RibH, possibly reflecting altered dissociation rates of pentamers from the assembled capsids. For the small low-mobile fraction observed (Figures 3D, 6C), we have proposed that these represent ribosome-associated assembly structures for capsids (Rotter et al., 2021). This fraction is not affected in its size by mutating K29 (Figure 6C).

#### Presence of RibT and Mutations in RibH^K29^ Affect Capsid Structure *in vitro*, Whereas Encapsulation of RibE Is Not Affected

**Figure 7:**
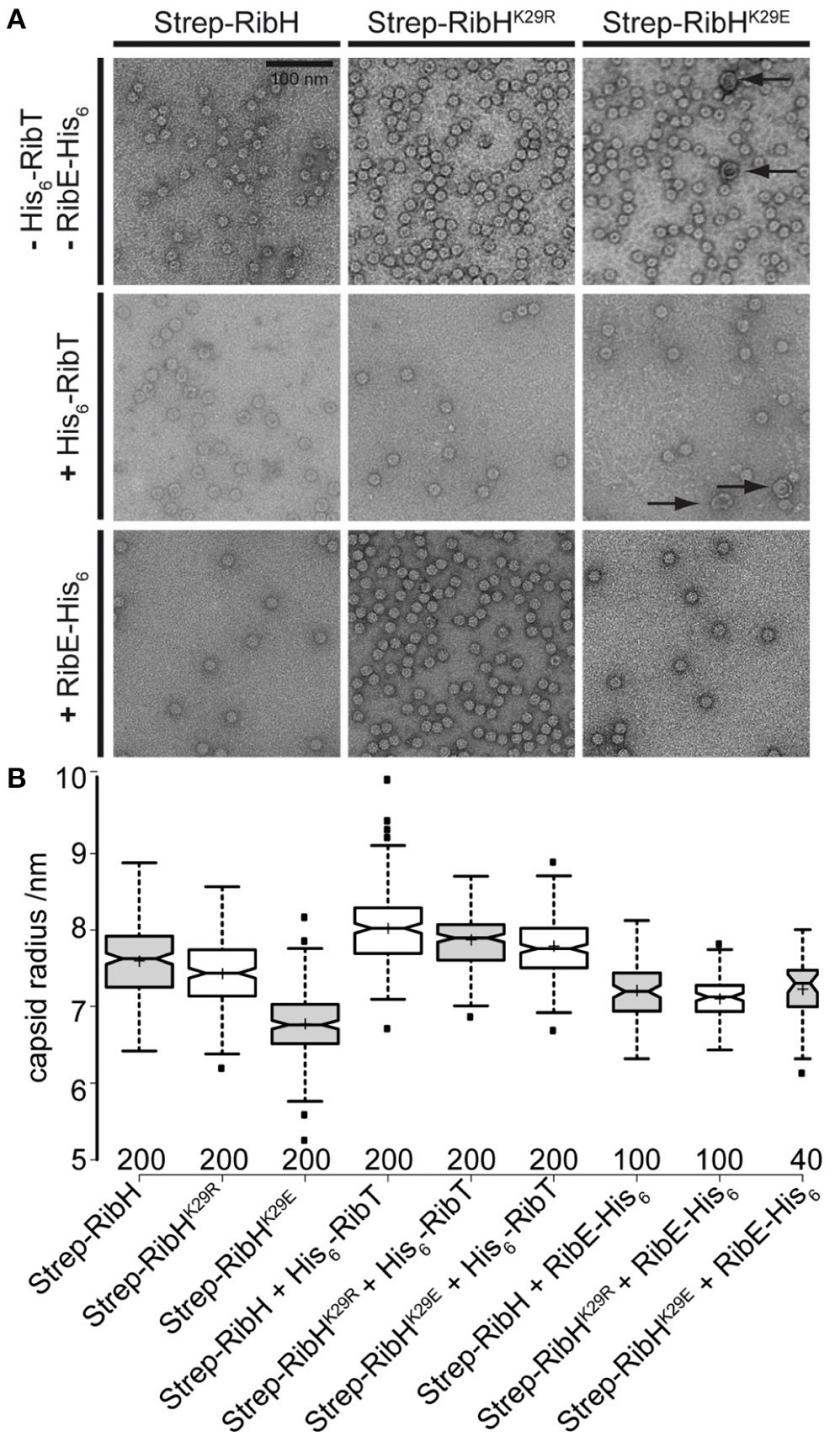
*In vitro* analysis of Strep-RibH and mutant capsids by TEM. **(A)** Electron micrographs of purified capsids either produced without any accessory Rib proteins (top), coproduced with His_6_-RibT from another compatible plasmid (middle), or coproduced with His_6_-RibE from the same plasmid (bottom). The scale bar (top left) is 100 nm and is valid for all electron micrographs shown. **(B)** Boxplots showing the statistical analysis of capsid radii as measured from electron micrographs using BoxPlotR (Spitzer et al., 2014). Medians are shown as black lines; notches represent the 95% confidence interval for each median. Sample means are shown as crosses, and the width of each box corresponds to the sample number, which is also given below each box. Outliers are shown as small, black squares.

In order to determine how the acetylation of K29 by RibT changes the structure of capsids formed by RibH, we decided to image and compare purified capsids by transmission electron microscopy (TEM) using negative staining. In the first step, we compared capsids solely formed by RibH, or RibH^K29E^, or RibH^K29R^. For this purpose, we produced the Strep-tagged version of all proteins in *E. coli*, followed by two-step purification using affinity chromatography and SEC. Elution profiles of Strep-RibH and of both mutants were very similar, eluting with apparent molecular masses of ~1 MDa in a calibrated Sepahacryl S400 SEC column (exemplarily shown for Strep-RibH in Supplementary Figure S8), indicating formation of stable 60-mer capsids *in vitro* in all three cases. Regarding the electron micrographs of Strep-RibH and mutant version (Figure 7A), particles were typically shaped as described for hollow RibH capsids without substrates (Ladenstein et al., 1988). When we measured radii of capsids, they were found to be ~7.6 nm (SD: ± 0.47 nm) for RibT, and 7.4 nm (SD: ± 0.46 nm) for RibH^K29R^, respectively (Figure 7B). However, capsids of mutant RibH^K29E^ revealed a smaller mean radius of ~6.8 nm (SD: ± 0.46 nm). Of note, we also found small number of considerably larger particles in electron micrographs, despite the use of SEC in which no characteristic peak(s) of higher MM were visible, indicating the formation of few much larger capsid structures. Those particles had a mean radius of a 14.2-nm (SD: ± 1.85 nm) characteristic for a 180-mer structure, which has been described before for native RibH capsids under certain *in vitro* conditions (Zhang et al., 2006). Because of their low abundance, we did not consider those larger capsids for the statistical analysis (Figure 7B); they likely belong to a different class of capsids characterized by a higher triangulation number (T = 3) in comparison to 60-mers (T = 1) (Zhang et al., 2006).

In the next step, we produced Strep-RibH and its two mutants, together with His_6_-RibT from another compatible plasmid. This way, we aimed at providing enough RibT activity under *in vivo* conditions for posttranslational modification of coproduced Strep-RibH in order to investigate capsid alterations.

**Figure 8:**
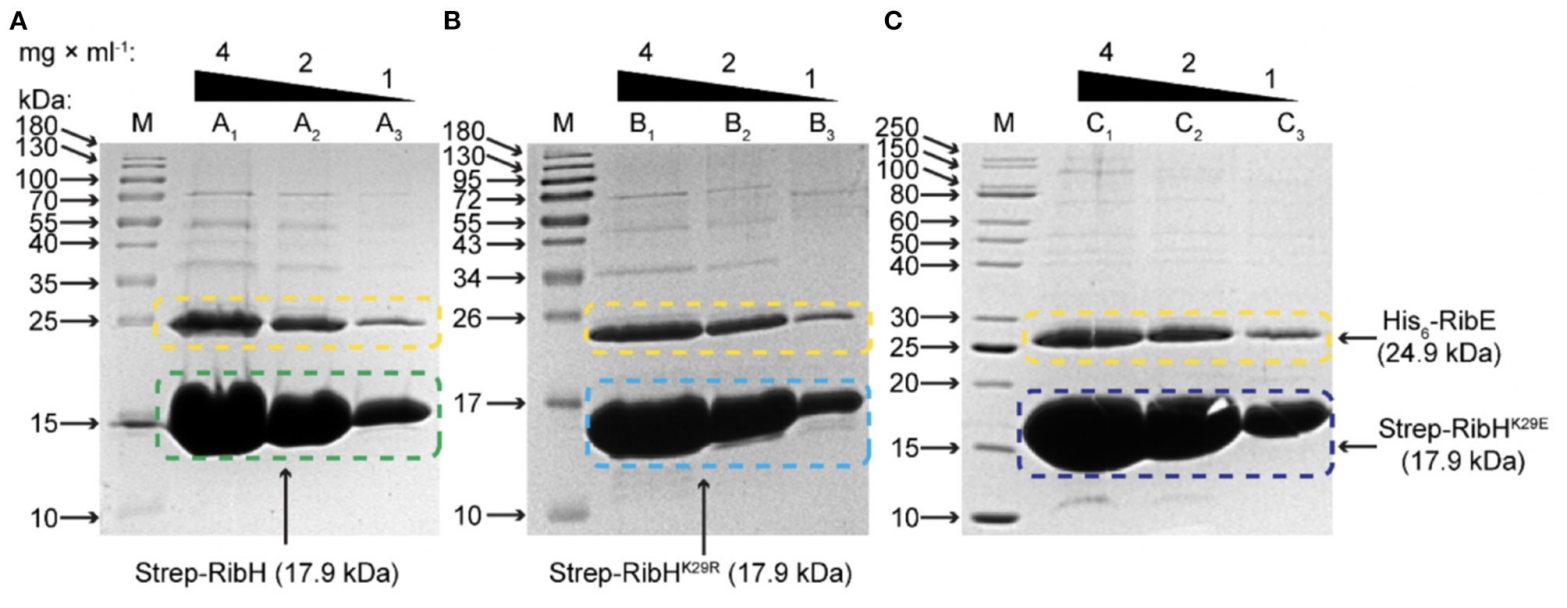
SDS-PAGE analysis for encapsulation of His_6_-RibE by Strep-RibH and its mutants Strep-RibH^K29R^ and Strep-RibH^K29E^. Proteins have been coproduced in *E. coli* BL21 Star and purified by Strep-Tactin affinity chromatography followed by SEC. The respective fractions from 60-mer peaks in SEC with apparent MM of ~1 MDa have been pooled, concentrated, and analyzed by SDS-PAGE in order to study encapsulation of His_6_-RibE. **(A)** Encapsulation of His_6_-RibE (24.9 kDa) by wt-like Strep-RibH (17.9 kDa). In lane M, the protein ladder has been applied, lanes A_1_, A_2_, and A_3_ denote for 4, 2, and 1 mg/ml of purified protein, respectively. **(B)** Encapsulation of His_6_-RibE (24.9 kDa) by mutant Strep-RibH^K29R^ (17.9 kDa). In lanes denoted with M, the protein ladder has been applied; lanes B_1_, B_2_, and B_3_ denote for 4, 2, and 1 mg/ml of purified protein, respectively. **(C)** Encapsulation of His_6_-RibE (24.9 kDa) by mutant Strep-RibH^K29E^ (17.9 kDa). In Lane M, the protein ladder has been applied; Lanes C_1_, C_2_, and C_3_ denote for 4, 2, and 1 mg/ml of purified protein, respectively.

Strep-RibH and its mutants were purified, as described before. In all cases, elution profiles did not indicate the presence of pentamers or 180 mers, but a small number of larger capsids were again found for mutant RibH^K29E^ (Figure 7A; black arrows). To our surprise, we found that the means of all capsid radii (Figure 7B) were similarly increased (~7.8–8 nm) when coproduced with RibT rather than without it (~6.8–7.6 nm). In case of Strep-RibH, we found a mean radius of 8. nm (SD: ± 0.47 nm), whereas radii of Strep-RibH^K29R^ and Strep-RibH^K29E^ were slightly less increased to 7.9 nm (SD: ± 0.33 nm) and 7.8 nm (SD: ± 0.38 nm), respectively. It is of note that the radius of mutant Strep-RibH^K29E^ increased the most (~7.8 nm) when RibT was coproduced compared to the sample with pure Strep-RibH^K29E^ (~6.8 nm). This result indicates that interaction of RibT and RibH may not solely be based on GNAT activity with substrate residue K29 but could also involve not yet identified residues. In a further approach, we coproduced Strep-RibH and its mutants together with His_6_-RibE (but not RibT) (Figure 7A). We found that Strep-RibH but also its mutants encapsulated His_6_-RibE as judged from SDS-PAGE analysis of isolated 60-mer peaks from SEC (Figure 8), indicating that mutations of K29 do not affect encapsulation abilities, and, furthermore, that RibT is not required for encapsulation of RibE. Interestingly, encapsulation of RibE by Strep-RibH and its two mutants revealed a similar effect in which capsids adopted a tighter conformation indicated by decreasing sizes of radii (7.1–7.2 nm) compared to both conditions before (Figure 7B).

Overall, *in vitro* analysis suggests that RibT interaction with the anticipated target residue K29 of RibH affects capsid architecture, but not encapsulation of RibE. Furthermore, RibT seems to influence overall capsid structure through additional, unknown interactions/activities.

## Discussion

We have analyzed the function of RibT, encoded by the last gene of the riboflavin biosynthesis operon in *B. subtilis*, using different *in vitro* and *in vivo* experiments. We show that RibT interacts with RibH, which forms the 60-mer shell of the heavy riboflavin (RF) synthase complex, and, with RibE, which is encapsulated as a trimer within the complex (Supplementary Figure S1). We provide evidence that RibT, shown to have structural properties of a GNAT acetyl transferase (Srivastava et al., 2018), acetylates residue K29, which lies in an interface of RibH pentamers, and shows that acetylation affects synthase capsid structure *in vitro*. These results suggest that riboflavin synthesis is also affected by an acetyl transferase acting on the final two steps, which occur as substrate channeling within the capsid. What may be the role of this process? We speculate that, besides, so far, unknown effects of acetylation of RibH on its own enzymatic activity, the acetylation state of K29 and possible additional residues in RibH affect (a) its subcellular localization and (b) its equilibrium with freely diffusing RibH pentamers.

In order to deal with the idea of subcellular localization achieved by anomalous diffusion, we have to consider the intricate rules of protein diffusion. Even in non-compartmentalized cells such as bacteria, diffusion of proteins cannot be simply explained by rules of Brownian motion, e.g., from a certain size upwards; there is no longer a marked decrease in diffusion coefficients but, rather, a plateau for mobility (Kumar et al., 2010; Parry et al., 2014). Despite of this, the modeling of diffusion *in vivo* using Brownian motion offers sufficiently important information to differentiate between mono-, oligo- or polymeric states of protein complexes (Rosch et al., 2018). Moreover, also, anomalous diffusion models, e.g., subdiffusion can be applied to yield information about a proteins motion, e.g., when a molecule shows frequent binding and unbinding events to a confined, lowly diffusing macromolecule (Oviedo-Bocanegra et al., 2021). For bacteria like *B. subtilis*, it has been shown that 70S ribosomes and polysomes accumulate at sites around chromosomes, which are compacted toward the cell middle as so-called nucleoids, indicating that transcription and protein biosynthesis are separated (Lewis et al., 2000; Mascarenhas et al., 2001). Interestingly, not only translating ribosomes are excluded from the nucleoid(s) “nucleoid occlusion: NO,” but even large enzymes, such as the large RF synthase complexes (Rotter et al., 2021). We have inferred that increased presence of Rib enzymes at the subpolar spaces due to low mobility may have benefits for the biosynthesis of a low-abundance cellular cofactor. In this work, we report that an acetyl transferase, RibT, affects the mobility of the enzyme complex heavy RF synthase. Higher levels of RibT led to a reduction or even loss of NO for the RibH/RibE complex, and additionally measurably increased its mobility. Of note, diffusion coefficients change with the diameter of proteins, so our measurements were at the detection limit, as changes due to acetylation do not strongly affect molecule dimensions, even for a 60(+3)-mer complex (Supplementary Figure S1).

Our data can be best explained by assuming that RibT sets up an acetylation state of RibH that optimizes disassembly of the heavy RF synthase. Single particle dynamics of RibH-mV can be explained by assuming a small population with extremely low diffusion coefficient, possibly an assembly state, a medium mobile fraction composed of the assembled complex, and a high mobility fraction comprising freely diffusing pentamers. We inferred that an equilibrium between complex and pentamers is important for optimal activity of RF synthase. By induced expression of *ribT* from an ectopic gene locus, we found an increase in the putative pentamer subpopulation and a decreased subpopulation considered as capsids. Lack of RibT had an opposing effect on RibH populations. Therefore, we hypothesize that RibT is responsible for the controlled release of pentamers from 60-meric RibH capsids. As a consequence of this, the diffusion and subsequent binding of the larger substrate (5-Amino-6-ribitylamino-2,4(1*H*,3*H*)-pyrimidinedione; ARIP) required for RibH may be facilitated. Contrarily, 3,4-Dihydroxy-2-butanone-4-phosphate (DHBP), which is less bulky compared to ARIP, is considered to easily pass the narrow channels of the complex (Kis and Bacher, 1995). In light of our results, the sense for the occurrence of a large number of non-encapsulated RibE in the cytoplasm can also be well explained (Bacher et al., 1980), because opening of the complex would lead to leaking of the product 6,7-Dimethyl-8-ribityllumazine (DMRL) formed by RibH, which is otherwise directly transferred to encapsulated RibE by a mechanism termed substrate channeling (Kis and Bacher, 1995). Furthermore, it is highly likely that loss of subunits also leads to a release of the final product RF, which is also too bulky to pass the existing channels at the five-fold symmetry axes (Ladenstein et al., 1994). Thus, we propose that RibT aids in shifting the equilibrium between assembled capsids toward freely diffusive pentamers in order to allow RF homeostasis in the cell. Furthermore, the release of pentamers allows for their high diffusion in the entire cytoplasm, which might facilitate substrate binding and, consequently, may lead to substrate-driven reassembly of the complex as it has been found in an *in vitro* study (Bacher et al., 1986).

Interestingly, reassembly of the full complex may preferentially happen at the crowded polar regions; due to the absence of the nucleoid(s), the effective concentrations of proteins in those regions are increased, which is thought to favor their interactions (Rotter et al., 2021). Indeed, deletion of *ribT* leads to a noticeable increase in (what we propose to be) assembled capsids and, consequently, in a decrease in fast-diffusing pentamers. Moreover, a decrease in confined molecules at cell poles was observed by deletion of *ribT*. Our view is supported by the fact that RF formation is reproducibly decreased in the *ribT* deletion mutant compared to a wt strain. These results underline that RibT is neither absolutely required to synthesize RF, nor to encapsulate RibE, which we show by *in vitro* experiments. It is tempting to speculate that RibT may help to provide the product from the heavy RF synthase under circumstances that demand fast release, e.g., for biosynthesis of proteins that require RF derivatives as cofactors like oxidoreductases, under stress conditions, which needs to be tested.

Our results suggest that residue K29 of RibH is a target of RibT, in agreement with earlier work (Carabetta et al., 2016). This residue is particularly attractive as a target for acetylation since it occupies the interface of three different pentameric subunits, which makes it present as a three-fold copy, and, additionally, it is easily accessible from the cytoplasmic lumen for RibT. It is conceivable that intermolecular protein-protein interactions at this interface may be substantially decreased by such a posttranslational modification. SPT of RibH-mV mutants shows that this residue seems to be particularly important for capsid integrity because its mutation to R (used as non-acetylatable mimicry of K) provides an increase in the respective diffusive subpopulation, whereas its mutation to E shows a decrease, which we associate with less stable capsids. Furthermore, mutation to R leads to a phenotype very similar to the *ribT* deletion mutant, showing a higher capsid subpopulation in SPT with respect to wt-like cells. Our *in vitro* analysis suggests that RibT does not interact exclusively with the anticipated target residue K29 of RibH but, furthermore, seems to influence overall capsid structure through other hitherto unknown interactions. Additionally, we can rule out that residue K29 of RibH plays a critical role in encapsulation, because RibE was still encapsulated even when K29 was mutated.

Under physiological growth conditions, the intracellular abundance of RibT is relatively low, at around ~50 copies per cell (Carabetta et al., 2016). By SPT of RibT-mV, we found the protein to be either freely diffusive, mainly over nucleoid regions, or confined at the cell poles. These findings indicate that RibT-mV binds the heavy RF synthase mostly at the sites of its higher abundance, which makes perfect sense.

In conclusion, our results point to a possible posttranslational mechanism that allows for RF homeostasis in the bacterial cell by a proposed exchange of RibH pentamers in the heavy RF synthase that might allow for product release. Clearly, RibT acetylates a large enzyme complex, affects its *in vivo* dynamics, and helps retain NO localization, which, to our knowledge, has not yet been reported in the literature.

## Materials and Methods

### Bacterial Strains and Growth Conditions

All bacterial strains used in this study are listed (Table 2). Bacterial strains were routinely grown using liquid lysogeny broth (LB) media (10 g/l tryptone, 5 g/l yeast extract, 10 g/l sodium chloride, pH 7.5) or solid LB-agar plates (liquid LB, 15 g/l agar). Molecular cloning of plasmids and their propagation was achieved using *E. coli* strain XL-1 Blue (Stratagene) as a host strain. Strains in this study were routinely cultivated in liquid LB media at 37°C under constant shaking (200 rpm), with addition of the respective antibiotics (Supplementary Table S3), ensuring selective pressure. Plasmid Mini Kit (Qiagen) was used for preparation of plasmids according to the manufacturer's instructions. Preparation and heat-shock transformation of competent *E. coli* strains were achieved by standard protocols (Sambrook and Russell, 2006a).

**Table 2:** Strains used in this study.

**Strain**	**Relevant genotype**	**Source or reference**
*E. coli* XL-1 Blue	*recA1 endA1 gyrA96 thi-1 hsdR17 supE44 relA1 lac* [F *proABlacIqZΔM15* Tn*10* (Tet^r^)]	Stratagene
*E. coli* BTH101	F-*cya-99araD139 galE15 galK16 rpsL1 (Str r)hsdR2 mcrA1 mcrB1*.	Euromedex
*E. coli* BL21 (DE3) Star	F-*ompThsdS*_B_ (r_B_-, m_B_-) *galdcm rne131* (DE3)	Invitrogen
*B. subtilis* PY79	Prototrophic derivative of *B. subtilis* 168	Laboratory stock
*B. subtilis* 168	*trpC2*	Laboratory stock
*B. subtilis* 168 Δ*ribT*	*trpC2*; Δ*ribT::kan*(Kan^r^)	BGSC: BKK23240
PY79 Δ*ribH*	Δ*ribH::kan*(Kan^r^)	Rotter et al., 2021
PY79 Δ*ribE*	Δ*ribE::kan*(Kan^r^)	Rotter et al., 2021
PY79 *ribH-mVenus*	*ribH-mVenuscat*(Cm^r^)	Rotter et al., 2021
PY79 *ribT-mVenus*	*ribT-mVenuscat*(Cm^r^)	This study
**Derivatives of PY79** ***ribH-mVenus*** **(transformed with plasmid** ***pSG1193-ribT*****)**		
PY79 *ribH-mVenusamyE*::P_Xyl_*-ribT*	*ribHmVenus cat* (Cm^r^) *amyE::P_*Xyl*_-ribTspc*(Spec^r^)	This study
**Derivatives of PY79** **Δ*****ribH*** **(transformed with plasmid** ***pSG1164-ribT-mV*****)**		
PY79 Δ*ribHribT-mVenus*	Δ*ribH::kanribT-mVenuscat*(Kan^r^, Cm^r^)	This study
**Derivatives of PY79** **Δ*****ribE*** **(transformed with plasmid** ***pSG1164-ribT-mV*****)**		
PY79 Δ*ribEribT-mVenus*	Δ*ribE::kanribT-mVenuscat*(Kan^r^, Cm^r^)	This study
**Derivatives of PY79 (transformed with chromosomal DNA of BKK23240)**		
PY79 Δ*ribT*	Δ*ribT::kan*(Kan^r^)	This study
**Derivatives of PY79** **Δ*****ribT*****(transformed with plasmid** ***pSG1164-ribH-mV*****)**		
PY79 Δ*ribTribH-mVenus*	Δ*ribT::kan:ribH-mVenuscat*(Kan^r^, Cm^r^)	This study
**Derivatives of PY79 (transformed with plasmids** ***pSG1164-ribH**^***K*29*R***^**-mV*** **or** ***pSG1164-ribH**^***K*29*E***^**-mV*****)**		
PY79 *ribH^*K*29*R*^-mVenus*	*ribH^*K*29*R*^-mVenuscat*(Cm^r^)	This study
PY79 *ribH^*K*29*E*^-mVenus*	*ribH^*K*29*E*^-mVenuscat*(Cm^r^)	This study

*B. subtilis* strains created in course of this study were derived from the prototrophic laboratory strain PY79, the auxotrophic wt strain 168 or its respective deletion mutants ordered from the Bacillus Genetic stock center (BGSC) (Koo et al., 2017). Strains were routinely grown in liquid LB media at 30°C (200 rpm) for overnight culturing, with addition of the respective antibiotics. For SPT microscopy, overnight cultures were diluted 75-fold in glass tubes with 1 ml of freshly prepared S7_50_ minimal growth media, containing glucose as a carbon source. In case of inducible expression by xylose using the xylose promoter (P_Xyl_), glucose was quantitatively substituted for the same amount of fracture in the growth medium, and expression of P_Xyl_ was induced with 0.1% (w/v) of xylose. Cultures were grown under constant shaking at 30°C till reaching an exponential growth phase (OD_600_:0.4-0.6) until samples of live cells were taken for SPT microscopy experiments. S7_50_ minimal media was freshly prepared according to published procedures (Jaacks et al., 1989).

### Plasmid Construction for Integrative Plasmids

All plasmid constructs in this study with respect to integration at the original gene loci were derived from *pSG1164-linker-mVenus* (Rotter et al., 2021), which was created from the integrative single-crossover plasmid *pSG1164* (Lewis and Marston, 1999). Integration at ectopic *amy E*locus was achieved using *pSG1193-linker-mVenus* (Rotter et al., 2021), which was created from *pSG1193* (Feucht and Lewis, 2001). To generate plasmids used to transform *B. subtilis*, we amplified the respective DNA sequences by PCR with dNTP (NEB) and Phusion High Fidelity DNA Polymerase (NEB) using genomic DNA of *B. subtilis* PY79 as a template, and using the corresponding oligonucleotides (Sigma-Aldrich) listed in Supplementary Table S5. PCR products were purified by gel extraction using QIAquick Gel Extraction Kit (Quiagen), successively digested with restriction enzymes *AvrII* and *ApaI* (NEB) at the specified optimal temperature, followed by heat inactivation of enzymes at 65°C for 20 min, and then purified using QIAquick spin columns (Qiagen). The plasmids *pSG1164-linker-mVenus* or *pSG1193-linker-mVenus* were digested accordingly and, furthermore, dephosphorylated by treatment with Calf intestine phosphatase (CIP; NEB), similarly purified as described before, subsequently ligated using T4 Ligase (NEB) with the respective linear DNA insert fragment in a 1:3 molar ratio, and subsequently transformed into *E. coli* XL-1 Blue, as described in Bacterial strains and growth conditions to yield the plasmids listed in Supplementary Table S4. Correct insert sizes were confirmed by analytical restriction digestion of the respective plasmid using *XbaI* (NEB), and correct insert sequences were verified by Sanger sequencing (Eurofins).

### Mutagenesis PCR

#### Construction of Mutant RibH Sequences RibH^K29R^ and RibH^K29E^

In order to generate the *ribH* mutant sequences *ribH*^*K*29*R*^ and *ribH*^*K*29*E*^, we applied the fusion PCR mutagenesis method. To construct desired mutations in DNA sequences, we used oligonucleotides *Fw ribH AvrII* and *Rev ribH 29K-R* or *Rev ribH 29K-E* to introduce the respective, desired base substitutions in the first PCR product of 112 bps in size, using chromosomal DNA of *B subtilis* strain PY79 as a template. In the next step, we similarly amplified a second PCR product of 397 bps in size using oligonucleotides *Fw ribH 29K-R* or *Fw ribH 29K-E* and *Rev ribH ApaI*. Both PCR products were constructed to have partial overlapping sequences to allow their annealing in final PCR. After purification of both desired PCR products using Gel-extraction (Quiagen), a final PCR was set up using both PCR products in equimolar concentrations and oligonucleotides *Fw ribH AvrII* and *Rev ribH ApaI* for amplification of the full-length PCR product (480 bps), carrying the desired substitutions. After the final step of purification using Gel-extraction, the resulting mutagenized PCR products were successively digested using restriction enzymes *ApaI* and *AvrII* to yield 397 bps inserts and were further treated as described before (Plasmid construction for integrative plasmids).

#### Construction of Mutant RibT Sequences RibT^C112A^ and Plasmid pET-28a-RibT^C112A^-His_6_

The construction of coding sequences for mutant RibT^C112A^ was achieved by PCR using plasmid *pET-28a-ribT-his*_6_ (5,609 bps, Supplementary Table S4) as a template and oligonucleotides *Fw ribT Nco*I, and *Rev ribT C112A Xho*I, which introduced the mutations at the 3'-end of the gene (Supplementary Table S5). The desired PCR product (387 bps) was digested using *Nco*IHF and *Xho*I for 2 h at 37°C, followed by heat inactivation of enzymes at 80°C for 20 min. The plasmid vector *pET-28a* (5,369 bps) was treated to yield a linear fragment of 5,231 bps, accordingly, and further dephosphorylated using CIP. Both linear fragments were purified by gel extraction, set up for ligation reaction in a 1:3 molar ratio of linearized plasmid and inserted using T4 ligase, and subsequently transformed into XL-1 Blue as described before. Single colonies from transformation plates were inoculated in liquid LB media with the addition of kanamycin for plasmid preparation. The correct insert sequences were verified by analytical restriction digestion and sanger sequencing to confirm the construction of the final plasmid *pET-28a-ribT*^*C*112*A*^*-his*_6_ (Supplementary Table S4).

### Strain Construction

Deletion strains that were used in the course of this study have been created by transformation of *B. subtilis* PY79 wt with chromosomal donor DNA of the respective 168 deletion strain (Table 2). Those 168 strains were obtained from the BGSC (Koo et al., 2017), and their chromosomal DNA was prepared using the phenol-chloroform extraction method (see preparation of chromosomal DNA). Transformation was achieved using an aliquot of an overnight culture from the respective PY79 strain. Overnight cultures were grown at 30°C with shaking (250 rpm) in liquid LB media. Using an appropriate aliquot, we inoculated 10 ml of freshly prepared liquid modified competence media (MCM) in a 200-ml shaking flask to have an initial optical density of 0.08–0.1 measured at 600 nm (OD_600_). MCM was prepared freshly according to published procedures (Zafra et al., 2012). The culture was grown in MCM at 37°C under constant shaking (200 rpm) till it reached the stationary growth phase indicated by an OD_600_ of 1.4–1.6. For transformation of plasmid or chromosomal DNA, we used an aliquot of 1 ml and added a total of 1 μg of either plasmid or chromosomal DNA; as a control, we used 1 ml of the same culture without any addition of DNA. Each culture was further incubated for two more hours at 37°C with constant shaking, followed by streaking out different amounts of culture aliquots onto fleshly prepared, solid LB-agar plates containing the appropriate antibiotics (Supplementary Table S3). To allow for growth of RF-deficient strains, we had to add minimal amounts of RF (1 μg/ml, Sigma-Aldrich) to all growth media used for their cultivation. Following incubation for 1–2 days, the resulting transformed colonies were selected for Kanamycin resistance and the PY79 phenotype in case of transformations with chromosomal DNA from 168, stored, and further used for transformation of integrative *pSG1164-linker-mVenus* or *pSG1193-linker-mVenus*-derived plasmids (Supplementary Table S4).

### Preparation of Chromosomal DNA

Chromosomal DNA from *B. subtilis* 168 strains used as templates for PCR or for transformation purposes was prepared using the phenol-chloroform extraction method (Green and Sambrook, 2017a). Following extraction, high-molecular DNA was subjected to precipitation by 1-propanol for concentration (Green and Sambrook, 2017b), subsequently washed with 70% ethanol (w/v), air dried, and suspended in appropriate amounts of a TE buffer (100-mM TRIS-HCl, 10-mM EDTA, pH 8.0).

### Amylase-Assay

Integration of fusion enzyme coding sequences into the *amyE* locus of *B. subtilis* was probed using amylase assay. For this purpose, we streaked single colonies from a transformation onto single wells that have been prepared with solid LB agar media, containing 1% (w/v) of starch and the respective antibiotics, if needed. These plates were incubated overnight at 30°C to allow for colony growth. As a positive control, we used untransformed PY79 wt cells. The next day, wells were covered with Lugol's iodine and incubated at 30°C for 5-10 min to allow for visual screening of non-starch hydrolyzing colonies.

### Determination of Protein-Protein Interactions Using Bacterial Adenylate Two-Hybrid Analysis

For analysis of interactions between RibH, RibT, and RibE, either one of the plasmids, e.g., *pRibHT25* with the fused T25 domain (Supplementary Table S4), was used to transform the *E. coli* reporter strain BTH101 (Table 2), followed by secondary transformation of any of the T18 domain containing plasmids, e.g., *pRibHT18* (Supplementary Table S4). A positive interaction will create a functioning adenylate cyclase that synthesizes cAMP and subsequently stimulates expression of *lacZ* in *E. coli*. Growing colonies were qualitatively analyzed for the development of blue color dependent on the reporter gene product β-galactosidase (LacZ) using an overlay assay with X-Gal staining. The control protein Zip (encoded on *pKT25-zip* or *pUT18C-zip*) was used as a negative control in all cases.

### Fermentative Growth, Growth Measurement, and Determination of RF Concentrations

If not otherwise indicated, *B. subtilis* strains were aerobically grown at 37°C in a 2x Spizizen's minimal medium (Spizizen, 1958), supplemented with 0.02% casamino acids, 2% (w/v) yeast extract, and 10% (w/v) glucose or in liquid LB. *B. subtilis* 168 (Saito et al., 1979) is a wild-type strain with regard to riboflavin biosynthesis and uptake and was used as a control. Riboflavin levels in the supernatant of aerobic *B. subtilis* cultures in the time course of growth was monitored as follows: an aliquot from the culture containing cells and a medium (500 μl) was combined with 465-μl 4 N NaOH and vigorously mixed for 1 min. The mixture was neutralized by adding potassium phosphate (1 M, pH 8.0) and centrifuged for 5 min at 13,000 x g at room temperature. Riboflavin in the supernatant was determined by using a standard procedure employing HPLC (Mack et al., 1998). Using the above-described protocol, the total amount of riboflavin (intracellular riboflavin and riboflavin present in the fermentation broth) was measured. For the statistical analysis of the data, Student's T-test was applied, comparing two unknown means based on independent samples.

### Fluorescence Detection of Fusion Enzymes

To detect fusion enzymes by in-Gel fluorescence, we created cell lysates of the respective strains and performed SDS-PAGE of those samples. For this purpose, *B. subtilis* strains were grown till reaching the exponential growth phase as already described (Bacterial strains and growth conditions). Cells have been repeatedly pelleted and washed in minimal media and subsequently stored as a frozen pellet. Next day, the pellets were suspended in a buffer (100-mM NaCl, 50°mM EDTA, pH 7.5) and DNAse; RNASe and lysozyme were added to yield final concentrations of 1 mg/ml. This suspension was incubated for 30 min at 37°C. An SDS-sample buffer was added to yield a ten-fold concentrated lysate. In order not to irreversibly quench mVenus-derived fluorescence for further imaging, the samples were not cooked as usually done. Those samples were finally loaded on 12% SDS-PAGE gel for electrophoretic separation for 1 h at 4°C using 50 mA and 120 V. The mVenus-derived fluorescence from fusion proteins was detected with a Typhoon TRIO Imager System (GE Healthcare) using excitation at 488 nm and optimal integration time.

### SDS-PAGE

SDS-PAGE was performed according to standard protocols (Sambrook and Russell, 2006b) using 15% polyacrylamide gels.

### Preparation of Live Cells for SPT Experiments

Strains of PY79 used for SPT were grown as described before. For imaging of single particles in live cells, 3 μl of cell culture suspension were spotted on clean coverslips (25 mm, Menzel) and covered using freshly prepared 1% (w/v) ultra-pure agarose pads. Those agarose pads were made by melting the appropriate amount of agarose in a fresh S7_50_ minimal medium and by further sandwiching ~80-μl melted agarose solution between two small 12-mm coverslips (Marienfeld). To avoid drying, pads were freshly prepared and kept humid until further use. The coverslips used for microscopy were intensively cleaned by ultra-sonication in Hellmanex II solution (2% v/v) for 30 min and intensively rinsed in distilled water, followed by a second round of ultra-sonication in double-distilled water until they were dried using high-pressure airflow.

### SPT Slimfield Microscopy

SPT experiments in this study were conducted using an Olympus IX-71. This microscope was equipped with a high numerical aperture (NA) Total internal reflection (TIRF) objective (UAPON × 100, Oil, NA = 1.49), which was coupled to a light-emitting diode laser LuxX 515-100 (515 nm/100 mW) to achieve excitation of fluorophores, which were detected using a back-illuminated Electron Multiplying Charge Coupled Device (EMCCD) iXon Ultra camera (Andor Solis). The laser beam was focused onto the back focal plane and operated during image acquisition with up to 2 mW (60 W/cm^2^ at the image plane). Using Andor Solis, 4.21 stream acquisitions of 1,500 frames having 13.76-ms interval times (12 ms integration time) were acquired from live cell samples. Those streams were equally cropped according to photobleaching curves to yield 1,000 frames, subsequently adjusted for pixel sizes of 100 nm and time increments using Fiji (Schindelin et al., 2012). Tracking of single particles was done using u-track 2.2 (Jaqaman et al., 2008). Trajectories were considered only for further analysis if they had a length of at least five steps. Data analyses were done using SMTracker 2.0 (Oviedo-Bocanegra et al., 2021).

### Recombinant Sequence Construction, Heterologous Protein Production, and Purification

Recombinant protein production was achieved using *E. coli* strain BL21 (DE3) Star (Table 2) as a host for transformation of *pASK-IBA7*-, *pET-28a*-, *pET-Duet-1*- or *pCDF-Duet-1*-derived plasmids (Supplementary Table S4). Cloning of plasmids and cryoconservation of the respective strains were achieved using *E. coli* strain XL-1 Blue as a host (Table 2). Correct insert sizes of all plasmids were verified by analytical restriction digestion, and correct sequences were verified using sanger sequencing (Eurofins). For a detailed description of all clonings and purification steps, please see Supplementary Data.

### Acetyl Transferase Assay

Purified, monodisperse proteins (Strep-RibH and mutant Strep RibH^K29E^) were probed as targets for enzymatically catalyzed acetylation by purified RibT-His_6_ using a fluorescence-based assay specific for Acetyl-CoA (AcCoA)-dependent acetyl transferases (ENZO Life sciences) in combination with a fluorescence microplate reader (TECAN Infinite 200). The fluorescence readout at 520 nm relies on excitation at 380 nm of a fluorescent adduct that is formed upon reaction with free CoA when detection solution was added. The reaction mixtures (containing AcCoA) were set up in black 96-well plates in duplicate according to the manufacturer's instructions using different concentrations (~10 μM, ~100 μM) of probed acceptor substrates (Strep-RibH or Strep-RibH^K29E^) and varying concentrations (25, 50, 100, 200 nM) of acetyl transferase RibT-His_6_ (or mutant RibT^C112A^-His_6_) for endpoint assays. Blank samples have been set up using only the respective acceptor substrates in a buffer. In case of kinetic endpoint assay, fixed concentrations of ~10 μM or ~100 μM acceptor substrate and 100 nM of acetyl transferase have been used. Chloramphenicol acetyl transferase (CAT) supplied by the manufacturer was used as positive control for acetylation of chloramphenicol in order to qualify the assay. As a negative control, we used bovine serum albumin (BSA) and RibT-His_6_ in kinetic endpoint assays. Following incubation at room temperature with gentle orbital shaking (100 rpm) for different time intervals (a total of 60 min with 10 min or 20 min time intervals), reactions were quenched by addition of ice-cold 1-propanol. After all reactions were quenched, detection solution was equally added, followed by development for 10 min at room temperature, avoiding incident light and shaking. Finally, the samples were excited using 380 nm, and fluorescence was detected at 520 nm. Data were recorded using 20-μs integration time and optimal gain.

### Electron Microscopy and Data Analysis

For imaging of purified Strep-RibH capsids and the respective mutants by transmission electron microscopy (TEM), we applied carbon-coated grids (400 mesh), which were lyophilized by glow discharging (PELCO easiGlow, Ted Pella, USA). Staining was done using 2% (w/v) uranyl acetate, which was spotted onto the grids together with 1 μl of 100-μg/ml protein solution in a buffer (50-mM sodium-phosphate, 150-mM NaCl, pH 7.2), followed by a short washing step using ultrapure H_2_O. Those samples were analyzed with a JEOL JEM-2100 transmission electron microscope using an acceleration voltage of 120 kV. For image acquisition, an F214 FastScan CCD camera (TVIPS, Gauting) was used. Image analysis was done using ImageJ (Schindelin et al., 2012). For the determination of capsid radii, well contrasted capsids have been selected to draw circular regions of interest, largely avoiding black contrast outlines. Radii have been calculated from the measured area using Excel 2016 (Microsoft), and values have been statistically analyzed using BoxPlotR (Spitzer et al., 2014).

### Localization Error Estimation From SPT Data

To account for differences in localization precision between compared SPT datasets, we calculated estimates for the average localization errors using the intersection point of the ordinate axis and the linear fit for five lag times in individual TAMSD plots for the respective SPT datasets. The average localization errors determined that way are listed for each SPT dataset used in this study (Table 3).

**Table 3:** Mean estimated localization errors and radii of confinement for each SPT dataset used in this study.

**Dataset**	**Trajectories (minimum 5 steps)**	**Estimated localization error from MSD y-axes interception (nm)**	Resulting radius of confinement used for analysis (nm) error*2.5
RibT-mV	1,792	68.51	171
RibT-mV (Δ*ribE*)	1,524	94.75	237
RibT-mV (Δ*ribH*)	955	90.27	226
RibH-mV	16,084	56.65	142
RibH-mV (Δ*ribT*)	16,261	57.61	144
RibH-mV *amyE*::*P*_Xyl_*ribT*	15,607	67.69	169
RibH^K29R^-mV	17,641	54.91	137
RibH^K29E^-mV	17,416	57.86	145

### SPT Heat Maps for Subcellular Analysis on Confined and Free Trajectories

Based on the radii of confinement (Table 3), we have developed trajectory heat maps, displaying trajectories of an SPT dataset that have been classified either as confined or freely diffusive in a normalized cell representation (1 μm × 3 μm), resembling a typical cell of *B. subtilis* in the mid-exponential growth phase. A trajectory is considered as confined if it has at least one dwell event for a given number of steps, which we fixed to be either three, six, or nine. This confinement can be total (a confined track for N steps), or absent (freely diffusive for N steps), leading to their classification.

## Data Availability Statement

The original contributions presented in the study are included in the article/Supplementary Material, further inquiries can be directed to the corresponding author.

## Author Contributions

DAOR performed SPT experiments, analyzed data, created constructs and strains, purified proteins, and wrote the manuscript. CK created constructs for BACTH and performed the BACTH assay, growth measurements, and RF quantification. CH helped in creating strains and constructs and performed SDS-PAGE analysis with in-gel fluorescence detection. NS purified proteins and performed an acetylation assay with CH and DAOR. AS helped in creating constructs and purified proteins for *in vitro* experiments. TH performed negative-staining and transmission electron microscopy of purified proteins. MM and PLG analyzed data, conceived of the study, and co-wrote the manuscript. All authors contributed to the article and approved the submitted version.

## Funding

This work was supported by the German Federal Ministry of Education and Research (BMBF)-funded coordinated graduate program NANOKAT and by the DFG-funded Transregio TRR 174.

## Conflict of Interest

DAOR was employed by BioNTech Manufacturing Marburg GmbH. CH was employed by BioSpringBiotechnolgie GmbH. The remaining authors declare that the research was conducted in the absence of any commercial or financial relationships that could be construed as a potential conflict of interest.

## Publisher's Note

All claims expressed in this article are solely those of the authors and do not necessarily represent those of their affiliated organizations, or those of the publisher, the editors and the reviewers. Any product that may be evaluated in this article, or claim that may be made by its manufacturer, is not guaranteed or endorsed by the publisher.
