# Supplementary material for: The Acetyltransferase RibT From Bacillus subtilis Affects in vivo Dynamics of the Multimeric Heavy Riboflavin Synthase Complex
Source: Front Microbiol. 2022 Apr 14;13:856820. doi: 10.3389/fmicb.2022.856820 (PMC9048828; doi:10.3389/fmicb.2022.856820)
Supplement: Supplementary file 1 [file Data_Sheet_1.pdf]

Supplementary data

Supplementary figures:

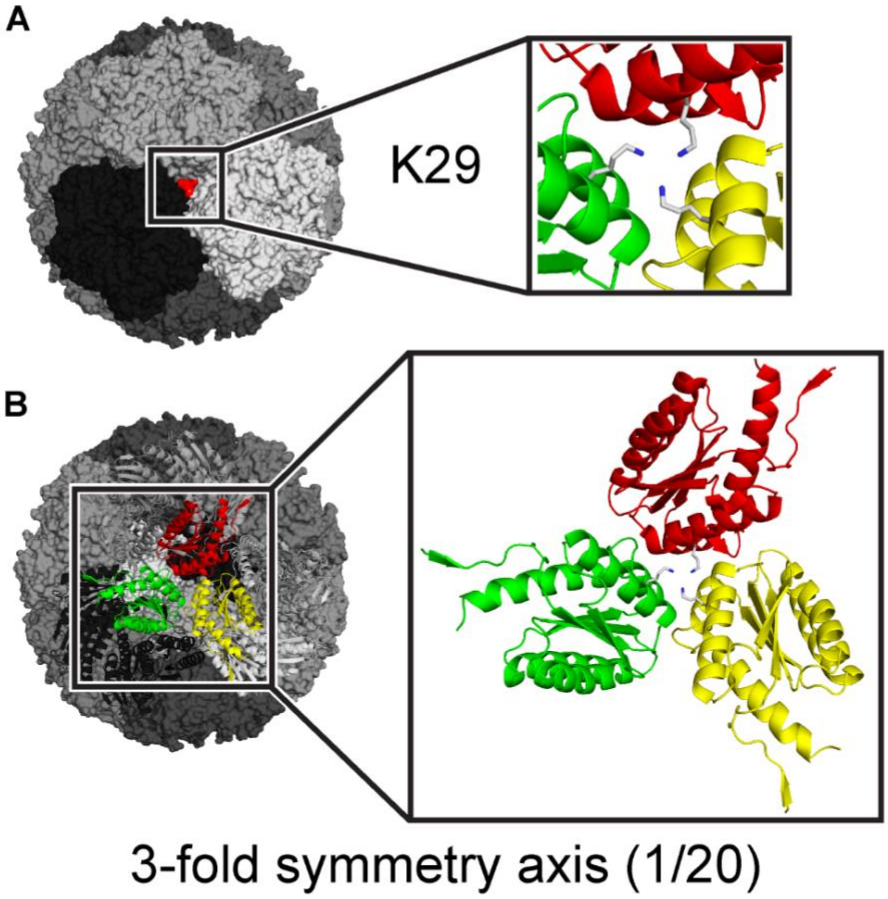

Figure S1: Structural analysis of K29 residue from RibH (A) 60-meric assembly of RibH according to PDB-file 1RVV (Ritsert et al., 1995). Pentameric subunits were colored as surfaces in different shades of grey using PyMOL (Schrodinger, 2015). **(A)** Surface model of 60-meric RibH highlighting one of the 20 fivefold symmetry axes in red where three residues of K29 originating from three pentameric units are in close proximity. The close-up view shows the same pentameric RibH units in differently colored cartoon style including the three K29 residues as organic side chain lines (Carbons colored in grey; Nitrogen colored in blue). **(B)** Surface model of 60-meric RibH in combination with three pentameric RibH units shown as cartoon style which attach each other at the same threefold symmetry axes as shown in A. The three monomeric RibH subunits that attach each other at the threefold symmetry axes in the center of the picture are colored in red, green or yellow and shown as cartoon style. The close-up view shows only the monomeric RibH subunits from three attaching pentameric units with their corresponding K29 residues as organic side chain lines (colors are the same as in A).

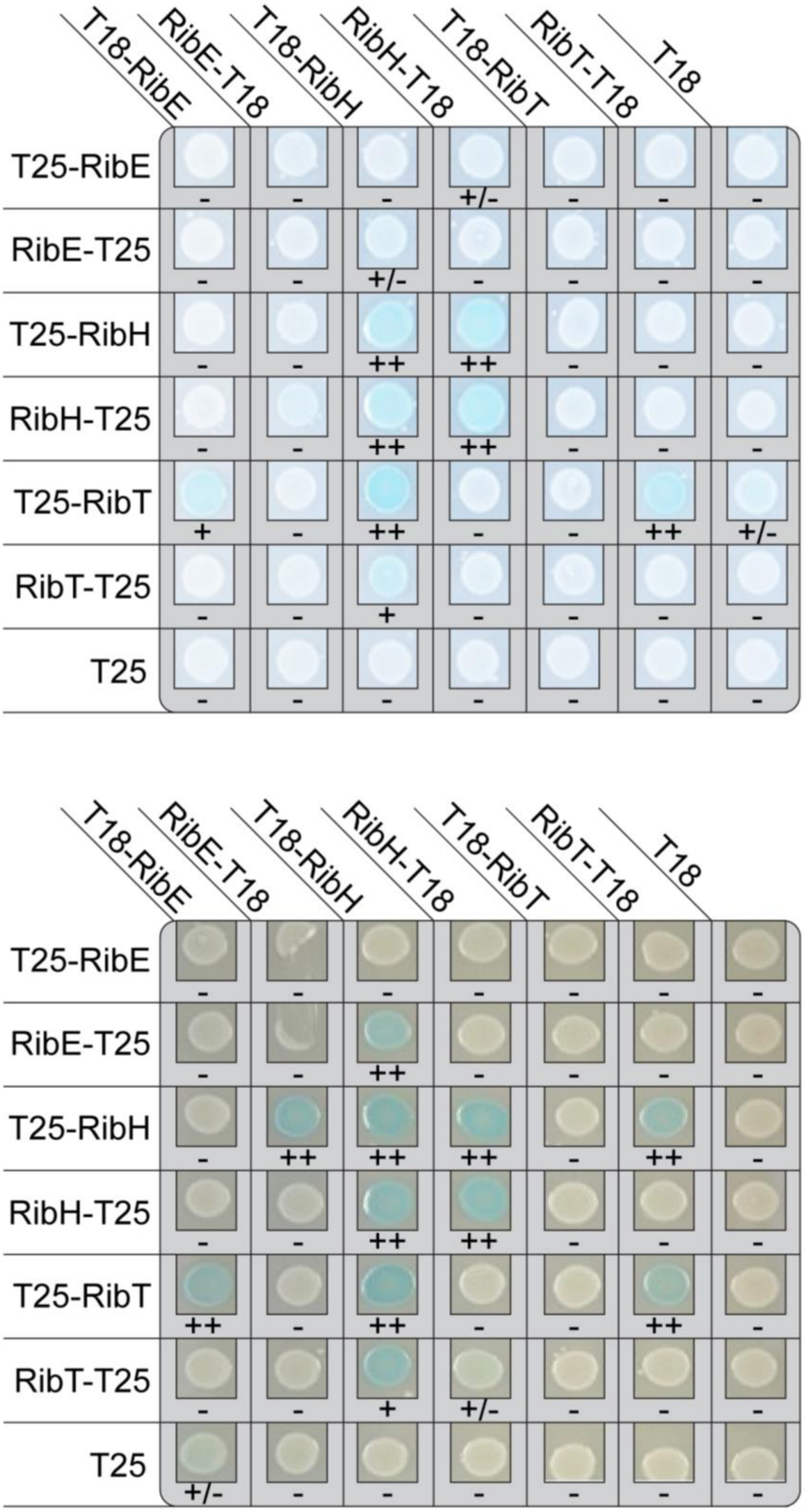

Figure S2: Two independent bacterial two-hybrid experiments. The BACTH assay has been carried out in triplicates. The third assay is shown in Figure 1. Shown are the colonies from plates, one day after inoculation, and evaluation of data, with dark blue (++) colonies, light blue (+) colonies, and ambiguous very light blue colored colonies considered as false positives (+/-).

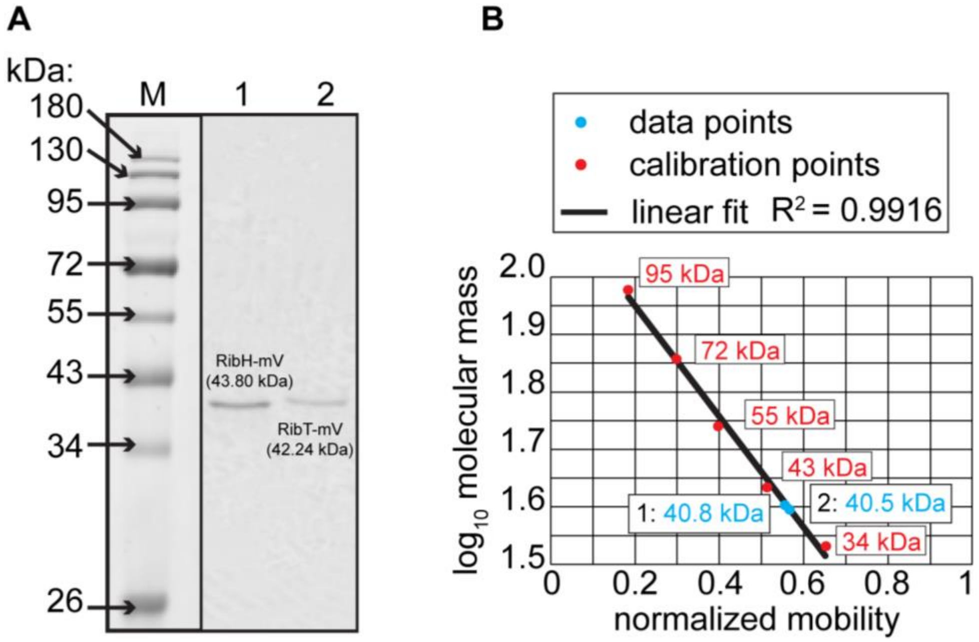

Figure S3: In-gel fluorescence detection of fusion enzymes RibH-mV and RibT-mV from SDS-PAGE analysis displaying full length fusion proteins. **(A)** The first lane (M) shows MM standard proteins (prestained), the other lanes refer to applied *B. subtilis* cell lysates from the respective strains producing RibH-mV (lane 1) or RibT-mV (lane 2) fusion proteins. The corresponding MM of each standard protein is indicated on the left. Theoretical MM of fusion proteins have been calculated according to their AA sequences and are indicated on top of each fluorescent protein signal **(B)** Semi-logarithmic plot displaying MM from standard proteins as log_{10} values (lane M; shown as red dots in the plot) used for linear calibration against their relative mobility. MM of fusion proteins (shown as light blue dots) have been calculated using the linear fit ($R^2 = 0.9916$) with the corresponding linear equation given here in brackets ($y = -0.9936x + 2.1422$).

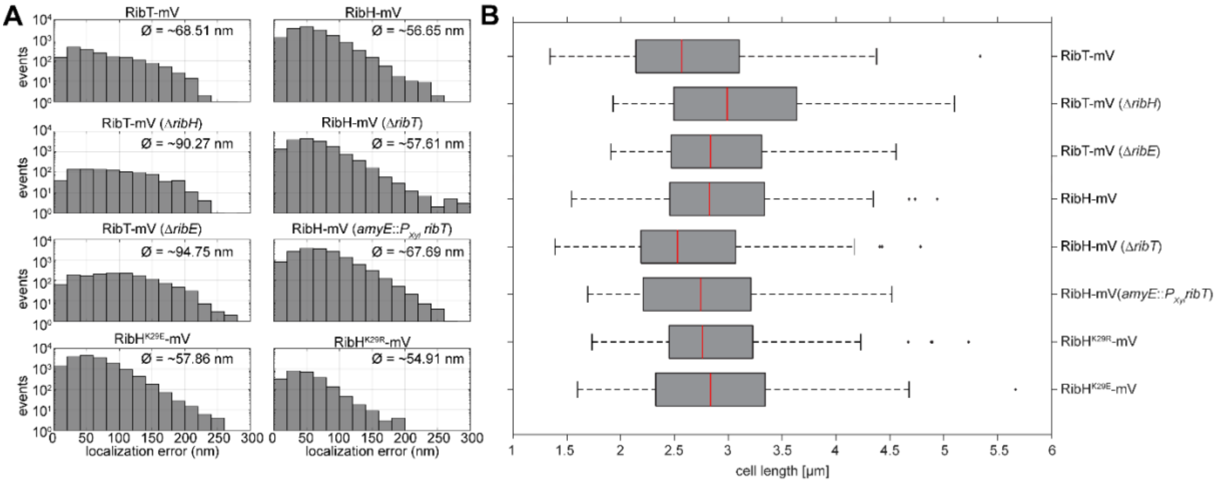

Figure S4: Localization error histograms and cell size boxplots for all SPT datasets in this study (A) Localization error histogram for all SPT datasets analyzed in this study. The mean localization error is indicated within each plot. **(B)** Cell size boxplots including all cells analyzed for the particular SPT datasets. The boxes denote for the 25% quantile to 75% quantile, the red lines denote for the medians, and outliers are shown as asterisk.

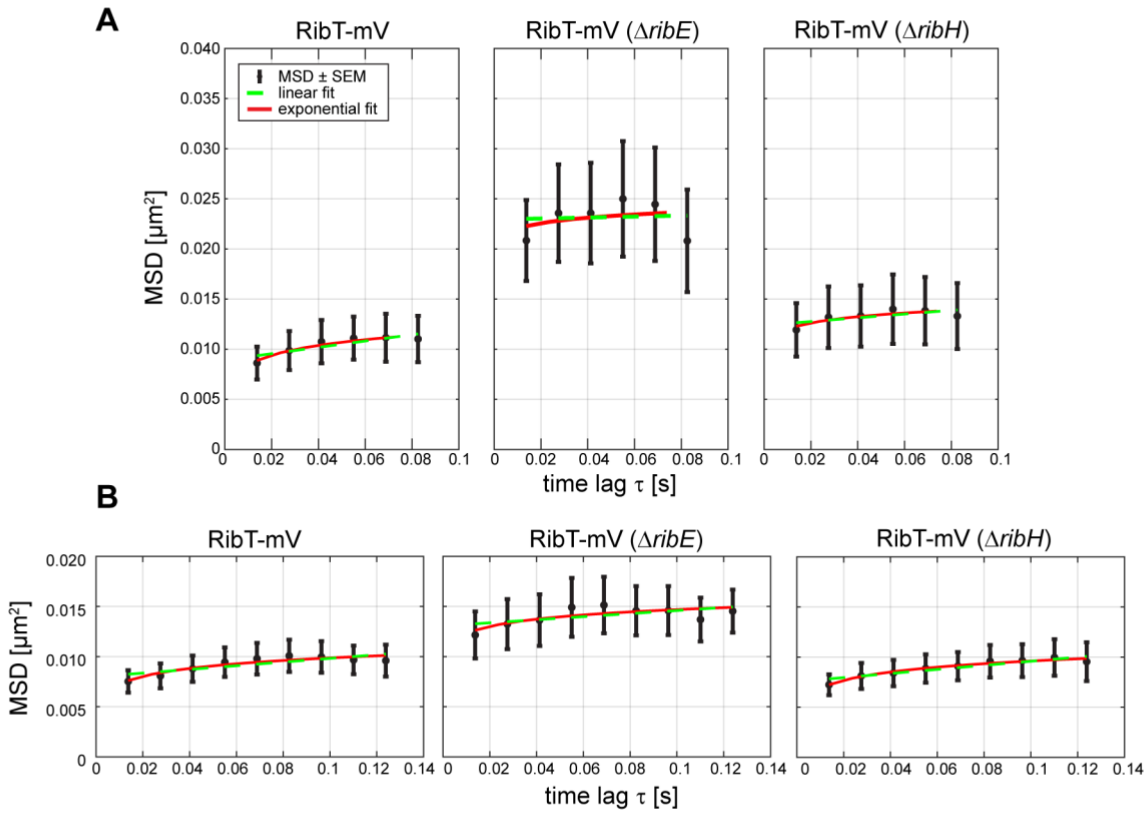

Dataset	N of confined steps considered	mode of diffusion	p-value (F-test result)	DC (µm²/s)	(α-value)
**RibT-mV**	6	subdiffusive	0.09142 (accepted)	0.0935	0.003822
	9	subdiffusive	0.03254 (accepted)	0.0918	0.003135
**RibT-mV (Δ*ribE*)**	6	Brownian	0.661 (rejected)	0.0012	1
	9	Brownian	0.114 (rejected)	0.0038	1
**RibT-mV (Δ*ribH*)**	6	Brownian	0.2493 (rejected)	0.0046	1
	9	subdiffusive	0.0129 (accepted)	0.0031	0.09724

Figure S5: EAMSD analysis of confined trajectories for RibT-mV under wt-like conditions, and with *ribE* or *ribH* being deleted. Trajectories classified as confined (according to the corresponding radii given in table 2) were pooled for a separate EAMSD analyses and F-test were performed to accept or reject subdiffusive behavior. The datasets analyzed are given on top of each plot. Diffusion coefficients (DC), respective p-values from each F-test, the number (N) of steps considered for analysis, and the resulting α-values are given in the table below **(A)** EAMSD plots of confined trajectories for RibT-mV under wt-like conditions, and with *ribE* or *ribH* being deleted analyzed for six consecutive steps of confinement. **(B)** EAMSD plots of confined trajectories for RibT-mV under wt-like conditions, and with *ribE* or *ribH* being deleted analyzed for nine consecutive steps of confinement.

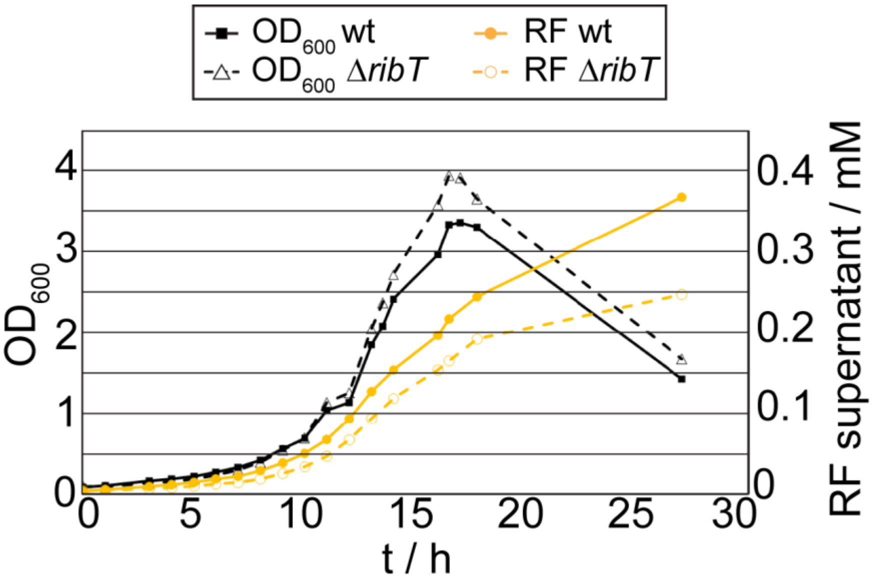

Figure S6: Fermentative growth and respective RF yield upon deletion of *ribT* in comparison to *B. subtilis* 168 wt strain. Displayed are RF yields (right y-axis) from the supernatant of lysed cells of either *B. subtilis* 168 wt (yellow filled circles) or mutant cells of 168 *ΔribT* (empty yellow circles). The corresponding optical density (OD_{600}; left y-axis) of the respective fermentative cultures are indicated and time points at which samples have been taken are shown over the x-axis.

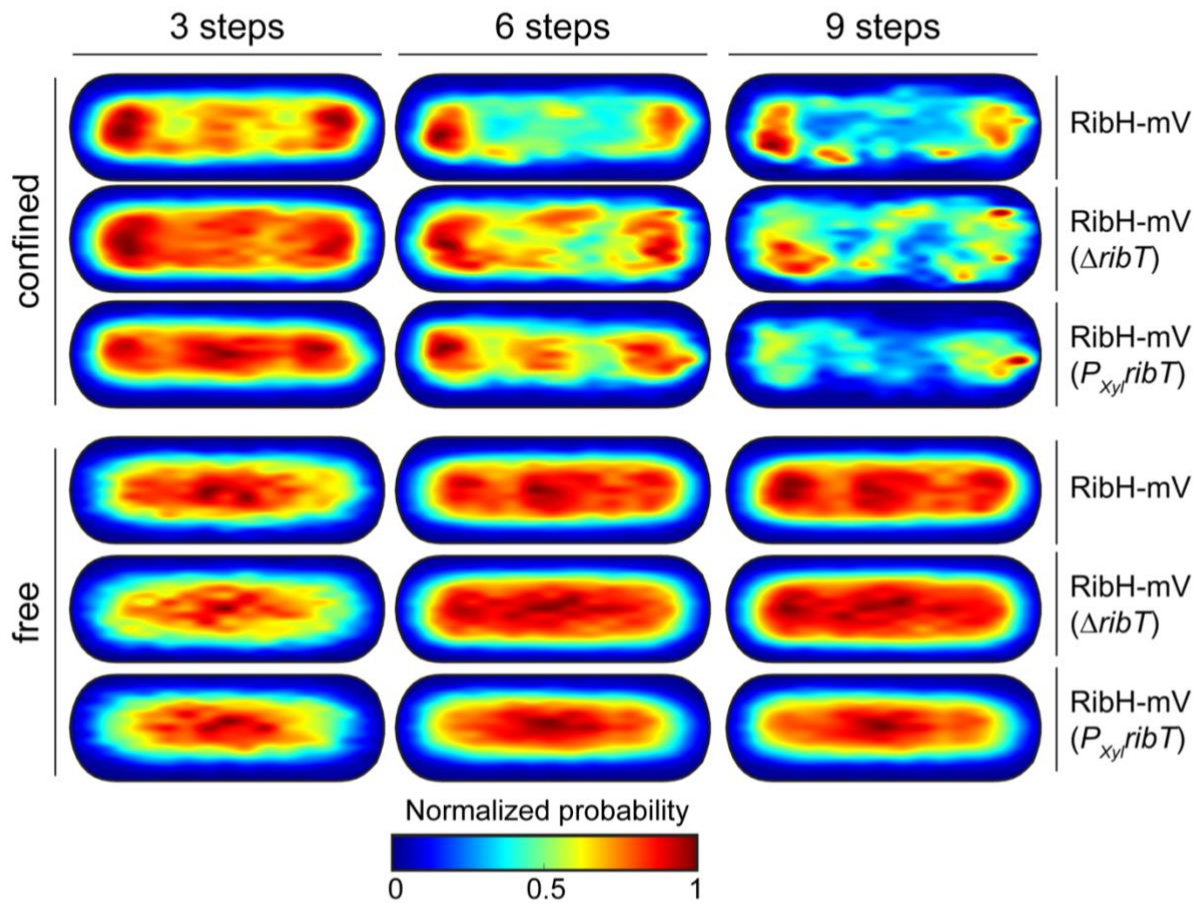

Figure S7: Subcellular analysis of confined and freely diffusing trajectories compared for RibH-mV (Δ*ribT*) with a strain expressing *ribT* from an ectopic locus and a wt like strain. Results are shown for different numbers of consecutive steps projected onto the cytoplasm of a normalized cell representation. Displayed are the results using the radii given in table 2 with three, six or nine consecutive steps of confinement.

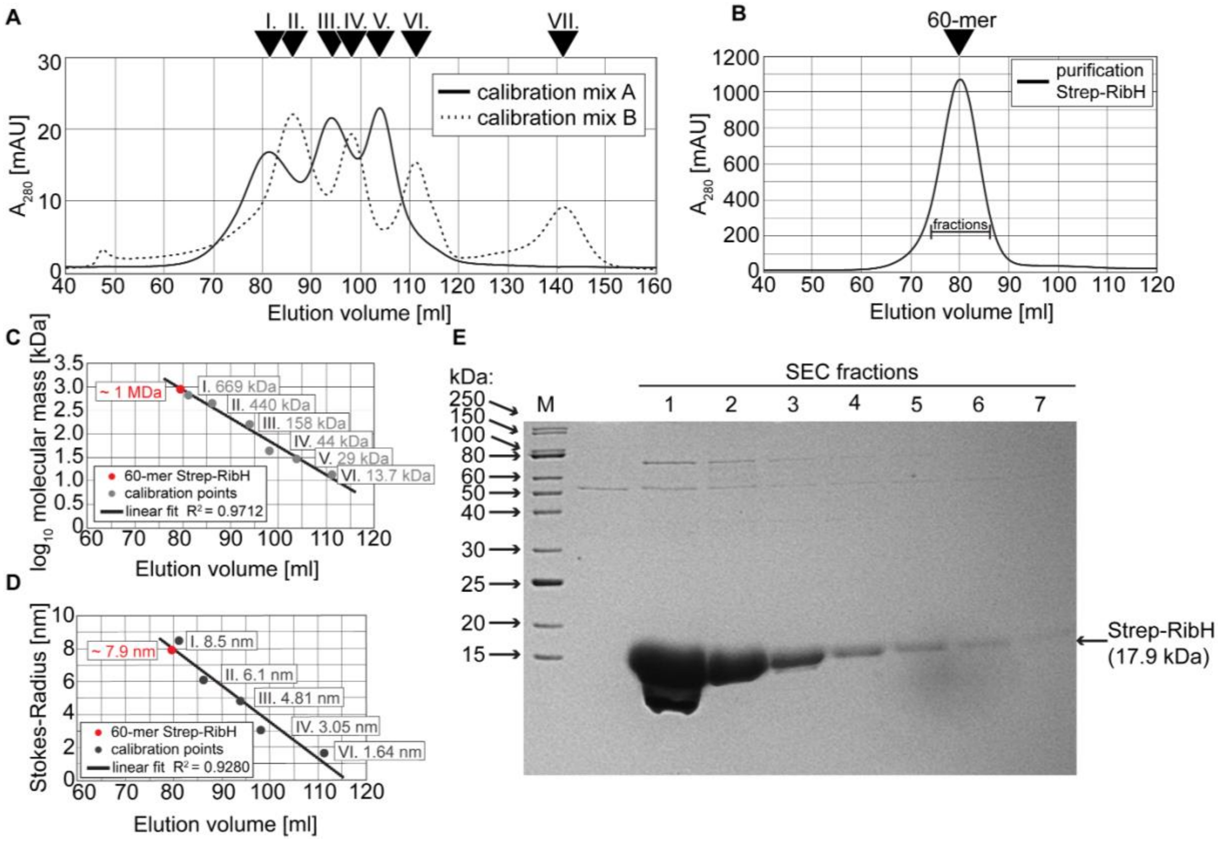

Figure S8: Purification of Strep-RibH 60-meric capsids. (A) Elution profiles of standard proteins used for calibration of the S400 Sephacryl column. The proteins used are indicated with roman numbers on top of the respective peak centers and are as follows: I. Thyroglobulin (Bovine thyroid), II. Ferritin (Horse spleen), III. Aldolase (Rabbit muscle), IV. Ovalbumin (Hen egg), V. Carbonic Anhydrase (Bovine erythrocytes), VI. Ribonuclease A (Bovine pancreas), and VII. Aprotinin (Bovine lung). **(B)** SEC elution profile of Strep-RibH (exemplary also for the SEC purification of Strep-RibH mutants). The peak center is highlighted on top of the SEC elution profile indicating the purification of 60-meric capsids. The SEC fractions that have been used for further *in vitro* analysis are indicated within the profile. **(C)** Semi-logarithmic plot displaying MM from standard proteins shown in A as $\log_{10}$ values versus their elution volume in SEC. MM of 60-meric capsids derived from Strep-RibH and its mutants have been determined using the linear fit of standard proteins (R^2 = 0.971). Please note that Aprotinin (VII.) has not been used for calibration according to MM. **(D)** Plot displaying the known Stokes-Radius of standard proteins shown in A versus their elution volume during SEC. Please note that Carbonic Anhydrase (V.), and Aprotinin (VII.) have not been used for Stokes-Radius calibration. **(E)** SDS-PAGE analysis of Strep-RibH fractions purified by SEC showing Strep-RibH as an example with an apparent MM of 17.9 kDa. The fractions analyzed by SDS-PAGE are also indicated in B.

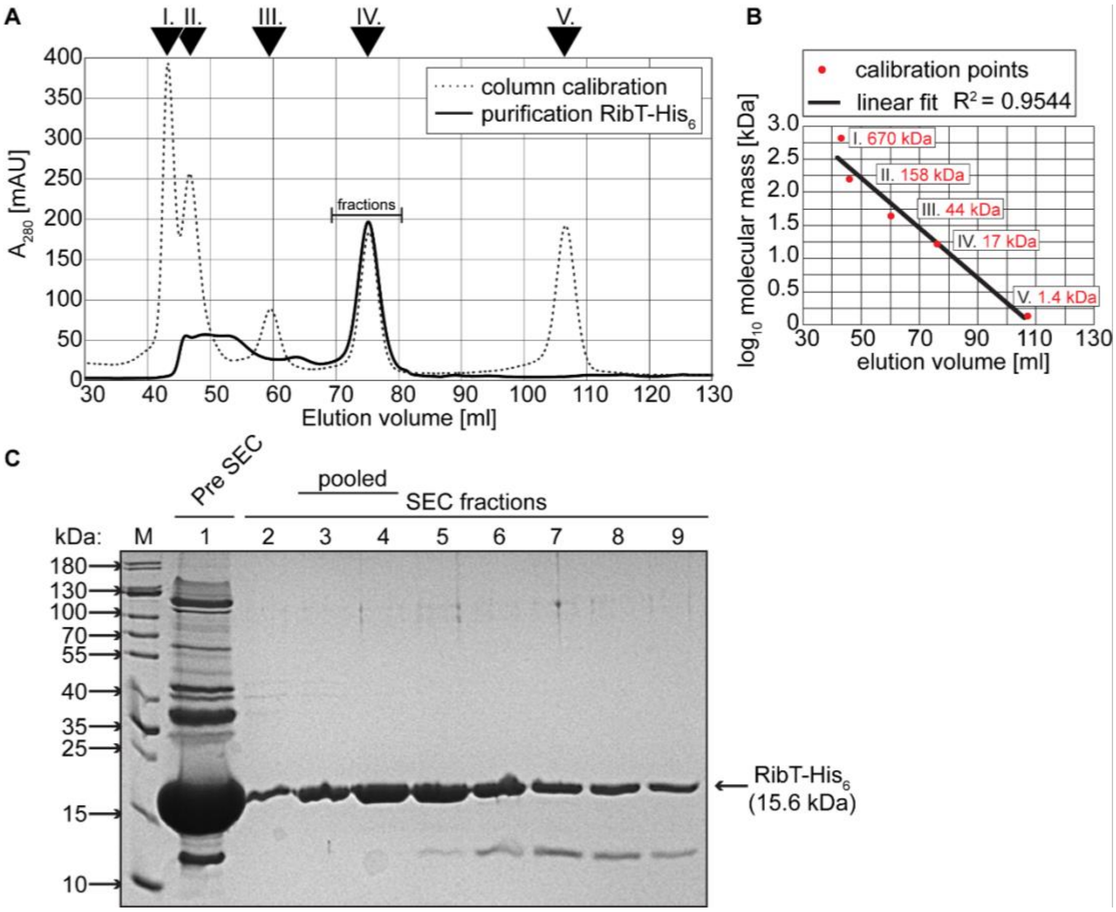

Figure S9: Purification of monomeric RibT-His$_6$ from *E. coli* BL21 Star by Ni^{2+}-NTA affinity chromatography and SEC. (A) SEC elution profile of RibT-His$_6$ which was crudely purified before SEC using Ni^{2+}-NTA affinity chromatography (shown in Panel C, lane 1), and elution profile of standards for column calibration. **(B)** Column calibration curve derived from the standard proteins shown in A. The semi-logarithmic plot displays MM from standards as log$_{10}$ values (shown as red dots) used for linear calibration versus their elution volume in SEC. Standards used for calibration are as follows: I. Thyroglobulin (bovine, 670 kDa); II. γ-Globulin (bovine, 158 kDa); III. Ovalbumin (chicken, 44 kDa); IV. Myoglobin (horse, 17 kDa); V. Vitamin B12 (1.35 kDa) **(C)** SDS-PAGE analysis of the purification process. Shown are the MM standard proteins (lane M), the crudely purified protein solution containing RibT-His$_6$ from affinity chromatography (lane 1), as well as the fractions analyzed from SEC according to the indicated width of the peak shown in A for RibT-His$_6$ (lane 2-9). The SEC fractions used for further *in vitro* experiments are indicated on top of the gel picture (pooled).

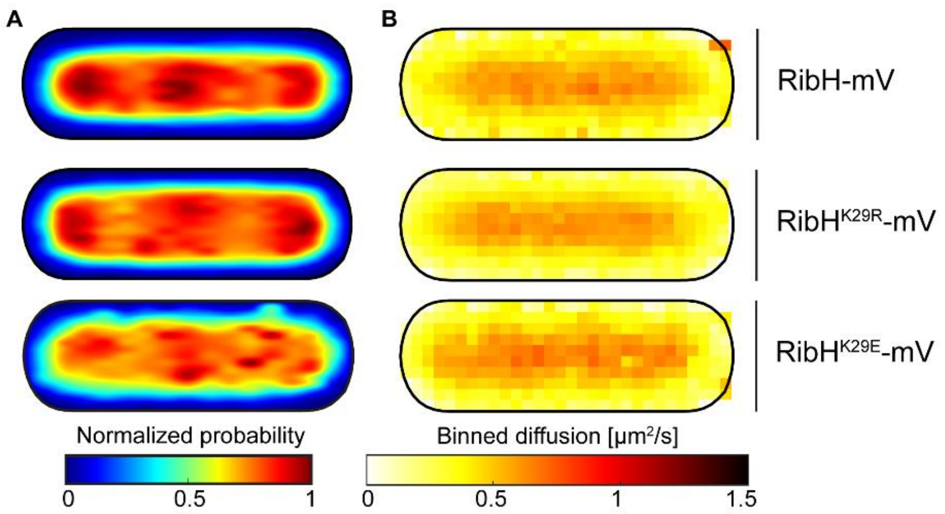

Figure S10: (A) Spot location heat-maps displaying the spatial distributions of trajectories for RibH-mV, RibH^{K29R}-mV, and RibH^{K29E}-mV. Trajectories were projected onto the two-dimensional cytoplasmic area of a normalized cell which resembles an averaged sized cell of *B. subtilis* in mid-exponential growth phase (1 µm × 3 µm). The likelihood of finding trajectories at a certain place in the cytoplasm is indicated by a color code from blue to red (indicated below). Signal intensities of spatial distribution maps have been normalized with each other. **(B)** Speed map representations of RibH-mV, RibH^{K29R}-mV, and RibH^{K29E}-mV displaying the spatial distributions of single step diffusion binned over areas of 0.1 µm^2 for normalized cells.

Supplementary tables:

Table S1: Summary of confinement analysis from all SPT data used in this study. Data sets are the same as in Tab. 2. For all analysis shown in here either three (a), six (b) or nine (c) steps of confinement were used.

**Strain relevant genotype** (R of confinement)	**Static**	**Mobile**	**% trajectories free vs. mixed** (Step distribution: free vs. confined)	**Dwell time** (1-comp. fit)	**Dwell times** (2-comp. fit)	**Transitions to dwell event** (% of trajectories)	**Transitions from freely diffusive to confined and *vice versa*** (% of trajectories with N steps)
**RibT-mV**<br><br>**(171 nm)**	a) 41.1%<br>b) 19.8%<br>c) 9.2%	a) 58.9%<br>b) 80.2%<br>c) 90.8%	a) 27% -31.9%<br>(29% - 71%)<br>b) 72.7% – 7.5%<br>(61% - 39%)<br>c) 88.1% - 2.7%<br>(78% - 22%)	a) 71 ± 1.5 ms (100%)	b) τ_1: 96 ± 3.6 ms (48 ± 8.9%)<br>τ_2: 150 ± 6.7 ms<br>(52 ± 8.9%)<br>c) τ_1: 120 ± 3.5 ms (18 ± 3%)<br>τ_2: 190 ± 2.4 ms<br>(82 ± 3%)	a) 1,391 (77.7%)<br>b) 501 (43.2%)<br>c) 214 (49.5%)	a) 541 (30.3%)<br>b) 111 (9.6%)<br>c) 36 (8.3%)
**RibT-mV, Δ*ribE***<br><br>**(237 nm)**	a) 31.7%<br>b) 10.3%<br>c) 3.7%	a) 68.3%<br>b) 89.7%<br>c) 96.3%	a) 24.9% - 43.4%<br>(33% - 67%)<br>b) 83.8% - 5.9%<br>(76%-24%)<br>c) 94.9% - 1.4%<br>(89% - 11%)	a) 57 ± 1.1 ms (100%)	b) τ_1: 92 ± 2.2 ms (67 ± 6.6%)<br>τ_2:160 ± 10 ms (33 ± 6.6%)<br>c) τ_1: 160 ± 4.1 ms (88 ± 6.2%)<br>τ_2: 350 ± 90 ms (12 ± 6.2%)	a) 1,215 (79.7%)<br>b) 251 (27.5%)<br>c) 79 (30.5%)	a) 624 (40.9%)<br>b) 85 (9.3%)<br>c) 18 (6.9%)
**RibT-mV, Δ*ribH***<br><br>**(226 nm)**	a) 37.9%<br>b) 14.9%<br>c) 6.9%	a) 62.1%<br>b) 85.1%<br>c) 93.1%	a) 29.3% - 32.8%<br>(33% - 67%)<br>b) 80.9% - 4.2%<br>(71% - 29%)<br>c) 92.5% - 0.6%<br>(84% - 16%)	a) 62 ± 1.3 ms (100%)	b) τ_1: 110 ± 3 ms (84 ± 6.6%)<br>τ_2: 220 ± 38 ms (16 ± 6.6%)<br>c) τ_1: 160 ± 7.6 ms (81 ± 13%)<br>τ_2: 300 ± 77 ms (19 ± 13%)	a) 697 (73%)<br>b) 182 (34.1%)<br>c) 72 (41.3%)	a) 306 (32%)<br>b) 39 (7.3%)<br>c) 7 (4%)
**RibH-mV**<br><br>**(142 nm)**	a) 12.9%<br>b) 4.1%<br>c) 0.9%	a) 87.1%<br>b) 95.9%<br>c) 99.1%	a) 23.3a% - 63.8%<br>(39% - 61%)<br>b) 78.4% - 17.5%<br>(78% - 22%)<br>c) 94.6% - 4.5%)<br>(92.8% - 7.2%)	a) 56 ± 0.8 ms (100%)	b) τ_1: 94 ± 0.5 ms (90 ± 2%)<br>τ_2: 160 ± 1 ms (9.6 ± 2%)<br>c) τ_1: 140 ± 1.6 ms (77 ± 5.4%)<br>τ_2:200 ± 12 ms (23 ± 5.4%)	a) 17,806 (110.7%)<br>b) 3,941 (30.8%)<br>c) 921 (13%)	a) 12,285 (76.4%)<br>b) 3,117 (24.3%)<br>c) 705 (10%)
**RibH-mV, Δ*ribT***<br><br>**(144 nm)**	a) 11.8%<br>b) 3.4%<br>c) 0.9%	a) 88.2%<br>b) 96.6%<br>c) 99.1%	a)24.4% - 63.8%<br>(41% - 59%)<br>b)80.6% - 16.1%<br>(81% - 19%)<br>c)95.4% - 3.7%	a) 55 ± 0.62 ms (100%)	b) τ_1:93 ± 0.63ms (91 ± 3.5%)<br>τ_2:140 ± 12 ms (9.2 ± 3.5%)<br>c) τ_1:130 ± 1.6 ms (75 ± 7%)<br>τ_2:180 ± 9.4 ms (25 ± 7%)	a) 17,703 (108.9%)<br>b) 3,514 (27.5%)<br>c) 786 (11.3%)	a) 12,726 (75.5%)<br>b) 2,884 (22.6%)<br>c) 575 (8.3%)

			(94.1% - 5.9%)				
**RibH-mV, *amyE*::P_{Xyl}*ribT*** <br>**(169 nm)**	a) 11.8%<br>b) 3.2%<br>c) 0.7%	a) 88.2%<br>b) 96.8%<br>c) 99.3%	a)23.3% - 64.9%<br>(39% - 61%)<br>b)81.3% - 15.4%<br>(82% - 18%)<br>c)96.1% - 3.2%<br>(95% - 5%)	a) 54 ±<br>0.65 ms<br>(100%)	b) τ_1:90 ± 1 ms (84 ± 7.9%)<br>τ_2:120 ± 9.1 ms (16 ± 7.9%)<br>c) τ_1:130 ± 1.5 ms (80 ± 7.2%)<br>τ_2:180 ± 13 ms (20 ± 7.2%)	a) 16,836 (107.9%)<br>b) 3,187 (25.9%)<br>c) 626 (9.6%)	a) 11,794 (75.6%)<br>b) 2,629 (21.4%)<br>c) 500 (7.7%)
**RibH^{K29R}-mV** <br>**(137 nm)**	a) 11.1%<br>b) 3.5%<br>c) 0.9%	a) 88.9%<br>b) 96.5%<br>c) 99.1%	a)23% - 65.9%<br>(40% - 60%)<br>b)78.3% - 18.2%<br>(80% - 20%)<br>c)94.5% - 4.6%<br>(93.1% - 6.9%)	a) 55 ±<br>0.37 ms<br>(100%)	b) τ_1:94 ± 0.4 ms (87 ± 1.8%)<br>τ_2:150 ± 5.8 ms (13 ± 1.8%)<br>c) τ_1:140 ± 0.77 ms (85 ± 2.3%)<br>τ_2:220 ± 10 ms (15 ± 2.3%)	a) 20,947 (118.7%)<br>b) 4,415 (30.6%)<br>c) 1,035 (12.2%)	a) 15,165 (86%)<br>b) 3,746 (26%)<br>c) 807 (9.5%)
**RibH^{K29E}-mV** <br>**(145 nm)**	a) 14.9%<br>b) 5.6%<br>c) 2.2%	a) 85.1%<br>b) 94.4%<br>c) 97.8%	a)24.4% - 60.7%<br>(38% - 62%)<br>b)78.7% - 15.7%<br>(77% - 23%)<br>c)93.5% - 4.3%<br>(90% - 10%)	a) 57 ±<br>0.32 ms<br>(100%)	b) τ_1:94 ± 0.38 ms (84 ± 0.94%)<br>τ_2:200 ± 5.7 ms (16 ± 0.94%)<br>c) τ_1:140 ± 0.59 ms (73 ± 0.95%)<br>τ_2:290 ± 4.6 ms (27 ± 0.95%)	a) 18,334 (105.3%)<br>b) 4,118 (30.5%)<br>c) 1,180 (16.1%)	a) 12,873 (73.9%)<br>b) 3,102 (23%)<br>c) 772 (10.5%)

Table S2: p-values derived from F-test for nested models (independent Rayleigh distribution modeling of JD distributions)

Strain<br>relevant genotype	p_{12}	p_{23}	Final model
ribT-mV	0	4.0767e-13	2 pop.
ribT-mV (Δ*ribE*)	0	0.00045001	2 pop.
ribT-mV (Δ*ribH*)	0	0.0029469	2 pop.
ribH-mV	0	0	3 pop.
ribH-mV (Δ*ribT*)	0	0	3 pop.
ribH-mV (*amyE*::P_{Xyl}*ribT)*	0	1.3528e-8	2 pop.
ribH^{K29R}*-mV*	0	0	3 pop.
ribH^{K29E}*-mV*	0	0	3 pop.

Table S3: Antibiotics used in this study

Antibiotic	Final concentration	Use
Ampicilin	100 µg/ml	Cultivation of XL-1 Blue, BTH101, and BL21 (DE3) Star strains transformed with *pSG-* , *pASK-IBA7-*or *pUT18-*, *pET-Duet1-* and *pUT18C-*derived plasmids
Chloramphenicol	5 µg/ml	Cultivation of PY79 strains with integrations at the original gene loci
Kanamycin	10 µg/ml	Cultivation of 168 deletion mutants, as well as, selection, and cultivation of the respective PY79 strains transformed with chromosomal DNA of 168 deletion mutants
Kanamycin	50 µg/ml	Cultivation of BTH101 transformed with *pKT25-* or *pKNT25-*derived plasmids, and cultivation of XL-1 Blue and BL21 (DE3) Star with *pET-28a-*derived plasmids
Spectinomycin	50 µg/ml	Cultivation of strains with ectopic integrations at *amyE* locus, and cultivation of XL-1 Blue or BL21 (DE3) Star strains transformed with *pCDF-Duet-1-*derived plasmids
Tetracyclin	20 µg/ml	Cultivation of XL-1 Blue strains

Table S4: Plasmids used in this study

Plasmid	Description / Reference	Use and Source
pSG1164-linker-mVenus	Integrative single crossover plasmid for original locus carrying C-terminal coding sequence for linker-mV	Rotter et al.,2021
pSG1193-linker-mVenus	Integrative double crossover plasmid for ectopic *amyE* locus integration carrying C-terminal coding sequence for linker-mV	Rotter et al.,2021
pSG1164-ribH-mVenus	Single crossover plasmid for *ribH*-mV integration to original locus	Rotter et al.,2021
pSG1164-ribT-mVenus	Single crossover plasmid for *ribT*-mV integration to original locus	This study
pSG1164-$ribH^{K29R}$*-mVenus*	Single crossover plasmid for $ribH^{K29R}$-mV integration to original locus	This study
pSG1164-$ribH^{K29E}$*-mVenus*	Single crossover plasmid for $ribH^{K29E}$-mV integration to original locus	This study
pSG1193-ribT	Integrative double crossover plasmid for ectopic *amyE* locus integration of *ribT* under control of inducible Xylose-Promotor (P_{Xyl})	This study
pKT25	Construction of pKT25-derived plasmids for BACTCH assay	Euromedex
pKTN25	Construction of pKTN25-derived plasmids for BACTCH assay	
pUT18	Construction of pUT18-derived plasmids for BACTCH assay	
pUT18C	Construction of pUT18C-derived plasmids for BACTCH assay	
pKT25-zip	Negative control for BACTH assay	
pUT18C-zip	Negative control for BACTH assay	
pKT25-ribE	BACTH assay of RibE-T25	This study
pKNT25-ribE	BACTH assay of T25-RibE	This study
pKNT25-ribH	BACTH assay of T25-RibH	This study
pKT25-ribH	BACTH assay of RibH-T25	This study
pKNT25-ribT	BACTH assay of T25-RibT	This study
pKT25-ribT	BACTH assay of RibT-T25	This study
pUT18-ribE	BACTH assay of T18-RibE	This study
pUT18C-ribE	BACTH assay of RibE-T18	This study
pUT18-ribH	BACTH assay of T18-RibH	This study
pUT18C-ribH	BACTH assay of RibH-T18	This study
pUT18-ribT	BACTH assay of T18-RibT	This study
pUT18C-ribT	BACTH assay of RibT-T18	This study
pET-28a	Expression plasmid for protein production offering N- or C-terminal His_6-tag	Novagen
pET-28a-ribT-his_6	Production of C-terminal His_6-tag RibT (RibT-His_6)	This study
pET-28a-$ribT^{C112A}$*-*his_{65}	Production of C-terminal His_6-tag mutant $RibT^{C112A}$ ($RibT^{C112A}$-His_6)	This study
pASK-IBA7	Expression plasmid for protein production offering N-terminal Strep-tag	IBA Lifesciences
pASK-IBA7-strep-ribH	Production of N-terminal Strep-tag RibH (Strep-RibH)	This study
pASK-IBA7-strep-$ribH^{K29E}$	Production of N-terminal Strep-tag mutant $RibH^{K29E}$ (Strep-$RibH^{K29E}$)	This study
pASK-IBA7-strep-$ribH^{K29R}$	Production of N-terminal Strep-tag mutant $RibH^{K29R}$ (Strep-$RibH^{K29R}$)	This study
pET-Duet-1	Expression plasmid for protein production from two target genes offering His-tag fusions	Novagen
pET-Duet-1-his_6*-ribE*	Used for cloning of *strep-ribH*, *strep-*$ribH^{K29R}$, and *strep-*$ribH^{K29E}$	This study
pET-Duet-1-his_6*-ribE-strep-ribH*	Coexpression of His_6-RibE and Strep-RibH	This study
pET-Duet-1-his_6*-ribE-strep-*$ribH^{K29R}$	Coexpression of His_6-RibE and Strep-$RibH^{K29R}$	This study
pET-Duet-1-his_6*-ribE-strep-*$ribH^{K29E}$	Coexpression of His_6-RibE and Strep-$RibH^{K29E}$	This study
pCDF-Duet-1	Expression plasmid for protein production of two target genes offering His-tag fusions (compatible *ori* with *pET-Duet-1* derived plasmids)	Novagen
pCDF-Duet-1-his_6*-ribT*	Coexpression of his_6*-ribT* with two other target genes from compatible plasmids derived from *pET-Duet-1*	This study

Table S5: Oligonucleotides used in this study

Oligonucleotide	5'-3' Sequence (Restriction recognition site)	Use
Fw XbaI ribE pKT25/pUT18C	TATGTCTAGAGATGTTTACAGGAATTATCGAAGAAACAGG	Construction of pKT25-ribE and pUT18C-ribE
Rev KpnI ribE pKT25/pUT18C	ATACTGGTACCCTAAAAGCCGTTTTCGCTTAAG	
Fw HindIII ribE pKTN25/pUT18	TGACTAAGCTTGATGTTTACAGGAATTATCGAAGAAAC	Construction of pKTN25-ribE and pUT18-ribE
Rev KpnI ribE pKTN25/pUT18	ATACTGGTACCCGAAAGCCGTTTTCG	
Fw XbaI ribH pKT25/pUT18C	TATGTCTAGAGATGAATATCATACAAGGAAATTTAGTTGGTACAGG	Construction of pKT25-ribH and pUT18C-ribH
Rev KpnI ribH pKT25/pUT18C	ATACTGGTACCTTATTCAAATGAGCGGTTTAAATTTGC	
Fw HindIII ribH pKTN25/pUT18	TGACTAAGCTTGATGAATATCATACAAGGAAATTTAGTTGGTAC	Construction of pKTN25-ribH and pUT18-ribH
Rev KpnI ribH pKTN25/pUT18	ATACTGGTACCCGTTCAAATGAGCGGTTTAA	
Fw XbaI ribT pKT25/pUT18C	TATGTCTAGAGATGTTAATTCGTTATAAAAAATCGTTTGAAAAGATTG	Construction of pKT25-ribT and pUT18C-ribT
Rev KpnI ribT pKT25/pUT18C	ATACTGGTACCTTAATTATTGTATGAAATGTCTTGATCCTGC	
Fw HindIII ribT pKTN25/pUT18	TGACTAAGCTTGATGTTAATTCGTTATAAAAAATCGTTTG	Construction of pKTN25-ribT and pUT18-ribT
Rev KpnI ribT pKTN25/pUT18	ATACTGGTACCCGATTATTGTATGAAATGTCTTGATCC	
Fw AvrII ribT	GTAGCCTAGGATGTTAATTCGTTATAAAAAATCGTTTG	Construction of pSG1193-P_{Xyl} ribT and pSG1164-ribT-mV
Rev ribT SpeI	CTACACTAGTGACTGACGTCATCCGTGAACGT	Construction of pSG1193-P_{Xyl} ribT
Rev ribT ApaI	TACGGGCCCATTATTGTATGAAATGTCTTGATC	Construction of pSG1164-ribT-mV
Fw ribH AvrII	GTACCTAGGATGAATATCATACAAGGAAATTTAGTTG	Construction of pSG1164-$ribH^{K29R}$-mV and/ or pSG1164-$ribH^{K29E}$-mV
Fw ribH 29K-R	TATTACGAGCAGGCTGCTGAGCGGAGCAG	
Fw ribH 29K-E	TATTACGAGCGAGCTGCTGAGCGGAGCAG	
Rev ribH 29K-E	CTGCTCCGCTCAGCAGCTCGCTCGTAATA	
Rev ribH 29K-R	CTGCTCCGCTCAGCAGCCTGCTCGTAATA	
Rev ribH ApaI	TACGGGCCCTTCAAATGAGCGGTTTAAATTTGCC	
Fw ribH BsaI	GTATAGGTCTCGGCGCAATATCATACAAGGAAATTTAGTTGGT	Construction of pASK-IBA7-derived plasmids
Rev ribH Stop BsaI	TAGTACGGTCTCATATCTTATTCAAATGAGCGGTTTAAATTTG	
Fw NcoI ribT	GTACCATGGATGTTAATTCGTTATAAAAAATCGT	Construction of pET-28a-ribT-His_6 and pET-28a-$ribT^{C112A}$-His_6
Rev ribT XhoI	GTACTCGAGATTATTGTATGAAATGTCTTGATCC	Construction of pET-28a-ribT-His_6
Rev ribT C112A XhoI	CTCGAGATTATTGTATGAAATGTCTTGATCCTGCTGACCTTGAGCACGTTCGAA	Construction of pET-28a-$ribT^{C112A}$-His_6
Fw ribE BamHI	GTACGGATCCGTTTACAGGAATTATCGAAGAAACAGGCA	Construction of pET-Duet-1-his_6-ribE
Rev ribE Stop HindIII	GTACAAGCTTCTAAAAGCCGTTTTCGCTTAAGAAGGCT	
Fw ribT BamHI	GTACGGATCCTAGCATTCGTTATAAAAAATCGTTTGAAA	Construction of pCDF-Duet-1-his_6-ribT
Rev ribT Stop AvrII	TACCCTAGGTTAATTATTGTATGAAATGTCTTGATCCTG	
Fw strep-ribH NdeI	GTACCATATGGCTAGCTGGAGCCACCCGCAGTTCGA	Construction of pET-Duet-1-his_6-ribE-strep-ribH, pET-Duet-1-his_6-ribE-strep-$ribH^{K29E}$, and pET-Duet-1-his_6-ribE-strep-$ribH^{K29R}$
Rev ribH Stop AvrII	TACCCTAGGTTATTCAAATGAGCGGTTTAAATTTGCCAT	

Table S6: Sequences, and molecular masses of purified, recombinant proteins, and their respective extinction coefficients used in this study

Protein (mm)	Sequence (tags in **bold**; mutations in ***bold and italic***)	Calculated extinction coefficient (Gasteiger et al., 2005) at 280 nm (theoretical P_I)
RibT-His_6 (15.6 kDa)	MLIRYKKSFEKIAMGLLSFMPNEKDLKQLQQTIKDYETDTDRQLFLWKEDEDIVGAIG VEKKDSEVEIRHISVNPSHRHQGIGKQMMDALKHLFKTQVLVPNELTQSFFERCQGQ QDQDISYNN**LEHHHHHH**	9970 M^{-1} cm^{-1} (6.18)
$RibT^{C112A}$-His_6 (15.6 kDa)	MLIRYKKSFEKIAMGLLSFMPNEKDLKQLQQTIKDYETDTDRQLFLWKEDEDIVGAIG VEKKDSEVEIRHISVNPSHRHQGIGKQMMDALKHLFKTQVLVPNELTQSFFER***A***QGQ QDQDISYNN**LEHHHHHH**	9970 M^{-1} cm^{-1} (6.18)
Strep-RibH (17.9 kDa)	**MASWSHPQFEKIEGR**NIIQGNLVGTGLKIGIVVGRFNDFITSKLLSGAEDALLRHGVD TNDIDVAWVPGAFEIPFAAKKMAETKKYDAIITLGTVIRGATTHYDYVCNEAAKGIAQ AANTTGVPVIFGIVTTENIEQAIERAGTKAGNKGVDCAVSAIEMANLNRSFE	15595 M^{-1} cm^{-1} (5.61)
Strep-$RibH^{K29R}$(17.9 kDa)	**MASWSHPQFEKIEGR**NIIQGNLVGTGLKIGIVVGRFNDFITS***R***LLSGAEDALLRHGVD TNDIDVAWVPGAFEIPFAAKKMAETKKYDAIITLGTVIRGATTHYDYVCNEAAKGIAQ AANTTGVPVIFGIVTTENIEQAIERAGTKAGNKGVDCAVSAIEMANLNRSFE	15595 M^{-1} cm^{-1} (5.61)
Strep-$RibH^{K29E}$ (17.9 kDa)	**MASWSHPQFEKIEGR**NIIQGNLVGTGLKIGIVVGRFNDFITS***E***LLSGAEDALLRHGVD TNDIDVAWVPGAFEIPFAAKKMAETKKYDAIITLGTVIRGATTHYDYVCNEAAKGIAQ AANTTGVPVIFGIVTTENIEQAIERAGTKAGNKGVDCAVSAIEMANLNRSFE	15595 M^{-1} cm^{-1} (5.19)
His_6-RibE (24.9 kDa)	**MGSSHHHHHHSQDP**FTGIIEETGTIESMKKAGHAMALTIKCSKILEDVHLGDSIAVN GICLTVTDFTKNQFTVDVMPETVKATSLNDLTKGSKVNLERAMAANGRFGGHFVSG HVDGTAEITRIEEKSNAVYYDLKMDPSLTKTLVLKGSITVDGVSLTIFGLTEDTVTISLIPH TISETIFSEKTIGSKVNIECDMIGKYMYRFLHKANENKTQQTITKAFLSENGF	6085 M^{-1} cm^{-1} (6.16)
His_6-RibT (16.0 kDa)	**MGSSHHHHHHSQDPS**IRYKKSFEKIAMGLLSFMPNEKDLKQLQQTIKDYETDTDRQL FLWKEDEDIVGAIGVEKKDSEVEIRHISVNPSHRHQGIGKQMMDALKHLFKTQVLVP NELTQSFFERCQGQQDQDISYNN	9970 M^{-1} cm^{-1} (6.18)

Supplementary Material and Methods

Construction of plasmid vectors encoding for Strep-fusion proteins

For construction of N-terminal Strep-tag fusion protein coding sequences chromosomal DNA of *B. subtilis* PY79 wt or mutant DNA of PY79 *ribH*^{K29E}*-mV* or PY79 *ribH*^{K29R}*-mV* (Tab. 3) were used as templates for PCR with oligonucleotides *Fw ribH BsaI* and *Rev ribH Stop BsaI* (Table S5). The resulting 495 bps fragments were digested using *BsaI* (NEB) for two hours at 37°C, and plasmid vector *pASK-IBA7* (3,246 bps) was digested, accordingly. After heat inactivation of restriction enzymes at 65°C for 20 min, and preparative gel extraction of restricted fragments, purified, linear, cohesive vector (3,166 bps) and inserts (466 bps) were ligated in a 1:3 molar ratio using T4-ligase (NEB), and subsequently transformed into XL-1 Blue. Finally, sequences of plasmid inserts for *pASK-IBA7-Strep-RibH*, *pASK-IBA7-Strep-RibH*^{K29E}, and *pASK-IBA7-Strep-RibH*^{K29R} (table S4) encoding for N-terminal Strep-tag II fusion enzymes Strep-RibH and mutants Strep-RibH^{K29E}, and Strep-RibH^{K29R}, respectively, were verified by Sanger sequencing (Eurofins) of plasmids prepared from cultures inoculated with single colonies from the according transformation plates.

Construction of pET-28a-ribT-his$_6$ *plasmids encoding for His-fusion protein* RibT-His$_6$

For construction of C-terminal His$_6$-tag coding sequences of RibT-His$_6$ chromosomal DNA of PY79 wt was uses as template for PCR with oligonucleotides *Fw NcoI ribT* and *Rev ribT XhoI*. The resulting 390 bps fragment was digested using restriction enzymes *NcoI* (High fidelity, HF) and *XhoI* for two hours at 37°C, followed by heat-inactivation at 80°C for 20 min. Plasmid vector *pET-28a* (5,369 bps) was digested, accordingly to yield a linear fragment with cohesive ends (5,231 bps). Both fragments were purified by gel-extraction and subsequently ligated and transformed as described before to finally yield plasmid *pET-28a-ribT his*$_6$ (Table S4).

Construction of pET-Duet and pCDF-Duet plasmids

For coproduction, encapsulation studies and transmission electron microscopy of Strep-RibH and mutant capsids Strep-RibH^{K29R}, and Strep-RibH^{K29E}, we constructed an N-terminal His-tagged fusion enzyme coding sequence for *his*$_6$*-ribE*. For this purpose, we used oligonucleotides *Fw ribE BamHI* and *Rev ribE Stop HindIII* (Table S5) in PCR with chromosomal DNA of PY79 as a template. The resulting PCR product (666 bps) was digested to yield a

cohesive fragment of 652 bps using restriction enzymes *BamHI* (HF, NEB) and *HindIII* (HF, NEB) for 2 h at 37°C. Plasmid vector *pET-Duet-1* (5420 bps; Novagen) was linearized, accordingly to yield a fragment of 5,383 bps. Following heat inactivation of restriction enzymes for 20 min at 80°C, and gel extraction of both compatible fragments, they were ligated, transformed and treated similarly as described before to yield the plasmid *pET-Duet-1-his*$_6$*-ribE* (Table S4). After insert sequencing the plasmid was further used for cloning of *strep-ribH* and the two mutant sequences *strep-ribH*^{K29R} and *strep-ribH*^{K29E}. The respective sequences were amplified from the constructed plasmids *pASK-IBA7-strep-ribH*, *pASK-IBA7-strep-ribH*^{K29R}, and *pASK-IBA7-strep-ribH*^{K29E} as templates for PCR using flanking oligonucleotides *Fw strep-ribH NdeI* and *Rev ribH Stop AvrII*. All three amplified DNA fragments (523 bps) were digested the same way using restriction enzymes *NdeI* (NEB) and *AvrII* (NEB) for 2 h at 37°C to yield fragments of 509 bps. Equally, we digested plasmid vector *pET-Duet-1-his*$_6$*-ribE* (Tab.5) to yield a linear fragment (5,900 bps) with cohesive ends for insertion of the second target gene and its mutant sequences. After heat inactivation at 65°C for 20 min of restriction enzymes, all fragments were purified by gel extraction and subjected to ligation reactions, and afterwards transformed into *E. coli* host strains as described before.

Protein production using pASK-IBA7 derived plasmids and purification of proteins

For the production of Strep-tag fusion enzymes readily constructed and sequenced *pASK-IBA7*-derived plasmids (Table S4) were transformed into in BL21 (DE3) Star host strains using standard protocols for heat-shock transformation (Sambrook and Russell, 2006a). The cell suspension was streaked onto solid, freshly prepared LB-Agar plates containing Ampicillin (Amp) for plasmid selection (Tab. 4). After incubation at 37°C for 16 h a single colony was selected for inoculation of a preculture in 10 ml tube of 2 ml liquid LB media containing Amp as described before. This preculture was grown overnight at 37°C under constant shaking (200 rpm) until the suspension was visibly turbid. Next day, the preculture was further used to inoculate a 500 ml shaking flask containing 50 ml of liquid LB media and Amp, as before. This culture was equally incubated and grown to an OD_{600} of ~0.3. Using this suspension, we inoculated five culture shaking flasks of 1 l volume with each one containing 100 ml LB media and Amp. These shaking flasks were equally incubated, as described before and bacteria were grown till reaching an OD_{600} of ~ 0.5 until anhydrotetracycline (2 mg/ml) was added to a final concentration of 200 ng/ml to induce target protein production. After induction cell

suspensions were allowed to grow further for three more hours until cells were harvested by centrifugation at 3000 g for 20 min at 4°C. For lysis of cells they were suspended in wash buffer (50 mM sodium-phosphate, 150 mM NaCl, 1 mM EDTA, pH 7.5) to yield ~ 50 ml cell suspension which was lysed using a cooled French press repeatedly until pressure decreased. The lysates were centrifuged at 10,000 g at 4°C for 1 h. The resulting supernatants were applied to a Strep-tactin Sepharose (IBA Lifesciences) column (1 ml) using a peristaltic pump with constant flow of 1ml/min in the cold-room. Strep-Tactin Sepharose columns were washed with two column volumes (CV) of wash buffer before elution was initiated by applying elution buffer (50 mM sodium-phosphate, 150 mM NaCl, 1 mM EDTA, 2.5 mM desthiobiotin) to the column. The resulting elution fraction was dialyzed against (50 mM sodium-phosphate, 150 mM NaCl, pH 7.2) for buffer exchange and concentrated simultaneously using Amicon Ultra-15 (10 kDa cut-off; Millipore) for centrifugation at 4000 g at 4°C for 30 min to yield ~10 mg/ml protein solution as detected by photometric measurement at 280 nm, and additionally by Bradford assay. Protein solutions were further subjected to size exclusion chromatography (SEC) using an equilibrated Sephacryl S400 Column (GE Healthcare) and Äkta Purifier FPLC system (GE Healthcare) at 4°C. SEC was performed using a precooled and degassed running buffer (50 mM sodium-phosphate, 150 mM NaCl, pH 7.2) with a constant flow rate of 1 ml/min. After their elution, eluted fractions were selected and analyzed by SDS-PAGE to probe for purity and finally pooled for further concentration using Amicon Ultra-4 (10 kDa cut-off; Millipore) to yield pure protein samples of ~8 mg/ml. Samples of pure protein solution were kept on ice in the cold-room until further use for *in vitro* experiments.

Protein production using pET-28a-ribT-his$_6$ and pET28a-ribT^{C112A}-his$_6$

For production of acetyltransferase RibT-His$_6$ or its mutant RibT^{C112A}-His$_6$ either plasmid vector *pET-28a-ribT-his$_6$ or pET-28a-ribT^{C112A}-his$_6$* have been used to transform *E. coli BL21* (DE3) *Star*. For protein production a singly transformed colony was selected for inoculation of 2 ml LB media with kanamycin (Tab. 4) in 10 ml tubes. This culture was incubated overnight at 37°C with shaking (200 rpm). The next day, culture suspension was diluted in 50 ml liquid LB media with kanamycin, and was further incubated as above till reaching an OD_{600} of approx. 0.4. Then, this culture was further used to inoculate a total of 500 ml of autoinduction media in a 5 l shaking flask. Autoinduction growth media contained 20 g/l tryptone, 5 g/l yeast extract, 5 g/l sodium chloride, 6 g/l Na_2HPO_4, 3 g/l KH_2PO_4, and sterile filtered 0.6% (w/v) glycerol,

0.05% (w/v) glucose, 0.2% (w/v) lactose, which have been added after autoclaving. The prepared autoinduction culture was allowed to grow overnight at 30°C with shaking (200 rpm). The next day cells were harvested by centrifugation of the cell suspension at 3000 g at 4°C. The resulting pellet was suspended in wash buffer (50 mM sodium-phosphate, 150 mM NaCl, 10 mM Imdiazol, pH 7.5), to yield ~ 50 ml cell suspension which was lysed using a cooled French press repeatedly until pressure decreased. The lysates were centrifuged at 10,000 g at 4°C for 1 h. The target proteins were purified from the resulting supernatants using a His-Trap HP 1 ml column and Äkta Prime (both GE Healthcare) in a first step. The supernatant was applied to the column with a constant flow of 0.75 ml/min at 4°C in the cold-room. After extensive washing using wash buffer at a flow rate of 1 ml/min, His-tagged proteins were eluted using elution buffer (50 mM sodium-phosphate, 150 mM NaCl, 150 mM Imidazole, pH 7.5) at a flow rate of 1 ml/min and the collected peak fractions of ~10 ml were subjected to buffer exchange and concentration using Amicon Ultra-15 (10 kDa cut-off; Millipore) for centrifugation at 4000 g at 4°C for 30 min with the addition of 20 ml SEC buffer to allow for buffer exchange (50 mM sodium-phosphate, 150 mM NaCl, pH 7.5). The concentrated protein solution (2 mg/ml) was further subjected to SEC using an equilibrated Superdex 75 prep grade column (GE Healthcare) and Äkta Purifier FPLC system (GE Healthcare) at 4°C. SEC was performed using a precooled and degassed running buffer (50 mM sodium-phosphate, 150 mM NaCl, pH 7.5) with a constant flow rate of 1 ml/min. After their elution, eluted protein fractions were further probed for purity, selected, concentrated as described before, and kept on ice as 1 mg/ml aliquots.

Protein coproduction using pET-Duet-1- and pCDF-Duet-1-derived plasmids

For coexpression of two recombinant genes compatible *pET-Duet-1-* and *pCDF-Duet-1-*derived plasmids have been used to transform *E. coli BL21* (DE3) *Star* host strains (Table S4). For protein production a singly transformed colony was selected for inoculation of 2 ml liquid lysogeny broth (LB) media with the respective antibiotic (Tab. 4) in 10 ml tubes. This culture was incubated overnight at 37°C with shaking (200 rpm). The next day, culture suspension was diluted in 50 ml liquid LB media with the respective antibiotics, and was further incubated as above till reaching an OD_{600} of approx. 0.4. Then, this culture was further used to inoculate a total of 500 ml of autoinduction media (as described before) in a 5 l shaking flask. The prepared autoinduction culture was allowed to grow overnight at 30°C with shaking

(200 rpm), and subsequently harvested and lysed as described before. The resulting lysates were centrifuged at 10,000 g at 4°C for 1 h. The target proteins were purified from the resulting supernatants similarly as described before in Protein production using *pASK-IBA7* derived plasmids and purification of proteins. The concentrations of purified protein samples were determined photometrical at 280 nm using the sequence-based calculated extinction coefficients (Tab. 7), and furthermore assayed using Bradford-reagent. For calibration we created a standard curve using different amounts of BSA (0.1-2 mg/ml) ensuring to have a linear range. Purified protein solutions were kept on ice in the cold-room until further use for *in vitro* experiments.

References:

Ritsert, K., Huber, R., Turk, D., Ladenstein, R., Schmidt-Base, K., and Bacher, A. (1995). Studies on the lumazine synthase/riboflavin synthase complex of Bacillus subtilis: crystal structure analysis of reconstituted, icosahedral beta-subunit capsids with bound substrate analogue inhibitor at 2.4 A resolution. J Mol Biol *253*, 151-167.

Schrodinger, LLC (2015). The PyMOL Molecular Graphics System, Version 1.8.
